# The impact of fertilization on ammonia-oxidizing bacteria and comammox *Nitrospira* communities and the subsequent effect on N_2_O emission and maize yield in a semi-arid region

**DOI:** 10.3389/fmicb.2023.1249668

**Published:** 2023-09-29

**Authors:** Setor Kwami Fudjoe, Lingling Li, Sumera Anwar, Shangli Shi, Junhong Xie, Frederick Kwame Yeboah, Linlin Wang

**Affiliations:** ^1^State Key Laboratory of Aridland Crop Science, Gansu Agricultural University, Lanzhou, China; ^2^College of Agronomy, Gansu Agricultural University, Lanzhou, China; ^3^Department of Botany, Government College Women University Faisalabad, Faisalabad, Pakistan; ^4^College of Grassland Science, Gansu Agricultural University, Lanzhou, China; ^5^State Key Joint Laboratory of Environment Simulation and Pollution Control, School of Environment, Beijing Normal University, Beijing, China

**Keywords:** nitrification communities, potential nitrification activity (PNA), keystone taxa, nitrogen use efficiency, competitive interaction

## Abstract

The control of nitrous oxide (N_2_O) emissions through nitrification and the optimization of maize yield are important in agricultural systems. However, within the semi-arid region, the impact of fertilization on the function of nitrification communities and its connection with N_2_O emissions in the rhizosphere soil is still unclear. Our study investigates the influence of fertilization treatments on the communities of ammonia-oxidizing bacteria (AOB) and the complete ammonia oxidizers of the *Nitrospira* known as comammox (CAOB) in a maize agroecosystem. Nitrous oxide production, potential nitrification activity (PNA), maize yield, and nitrogen use efficiency (NUE) were determined for the same samples. The fertilizer treatments included a control group without fertilization (NA), inorganic fertilizer (CF), organic fertilizer (SM), combined inorganic and organic fertilizer (SC), and maize straw (MS). The SC treatment indicated a lower cumulative N_2_O emission than the CF treatment in the 2020 and 2021 cropping seasons. The AOB community under the CF, MS, and SM treatments was predominantly composed of *Nitrosospira* cluster 3b, while the SC treatment was associated with the comammox *Nitrospira* clade A.1 lineage, related to key species such as Ca. *Nitrospira inopinata* and Ca. *Nitrospira nitrificans*. Network analysis demonstrated a positive potential for competitive interaction between hub taxonomy and distinct keystone taxa among AOB and comammox *Nitrospira* nitrifiers. The structural equation model further revealed a significant positive association between AOB nitrifiers and N_2_O emission, PNA, soil pH, SOC, ${\text{NO}}_{3}^{-}$-N, and DON under organic fertilization. The keystone taxa in the comammox *Nitrospira* nitrifier and network Module II exhibited a positive correlation with maize productivity and NUE, likely due to their functional activities stimulated by the SC treatment. It is noteworthy that the AOB community played a more significant role in driving nitrification compared to the composition of comammox *Nitrospira*. Collectively, combined inorganic and organic fertilizer (SC) treatment exhibits high potential for reducing N_2_O emissions, enhancing maize productivity, increasing NUE, and increasing the sustainability of the nitrogen dynamics of maize agroecosystems in the semi-arid Loess Plateau.

## Introduction

Excessive fertilization has raised numerous environmental and ecological concerns, including soil acidification, increased nitrous oxide (N_2_O) gas emissions, and water pollution (Hink et al., 2018; Fudjoe et al., 2021). N_2_O is a potent greenhouse gas with a global warming potential of ~300× that of carbon dioxide, which also poses a significant threat to the stratospheric ozone layer and overall climate stability (Schmidt et al., 2019; Chataut et al., 2023). Excessive nitrogen-based fertilization (NBF) continues to contribute to substantial levels of N_2_O emissions from agroecosystems. In 2015, agricultural soils accounted for ~55% of global N_2_O emissions, a proportion projected to increase to 59% by 2030. Therefore, enhancing nitrogen use efficiency (NUE) in agriculture has become crucial for mitigating rising N_2_O emissions (Cardenas et al., 2019; Govindasamy et al., 2023). Improving NUE can also lead to increased crop yields, reduced fertilizer costs, and improved soil health. To maximize efforts in mitigating N_2_O emissions for sustainable soil ecosystems, the use of combined approaches such as the integration of inorganic and inorganic fertilizers has been recommended (Hink et al., 2018; Yang et al., 2018; Wang et al., 2019).

Nitrification is the key stage in the nitrogen cycle, converting ammonia (NH_3_) into nitrite (${\text{NO}}_{2}^{-}$) and then nitrate (${\text{NO}}_{3}^{-}$), thus making nitrogen more mobile to plants and microbes. However, this process can lead to nitrogen loss through ${\text{NO}}_{3}^{-}$ leaching, N_2_O, and N_2_ emissions (Yu et al., 2019; Wang et al., 2020; Sun et al., 2021). With the discovery of complete ammonia oxidizers of the *Nitrospira* known as comammox (CAOB), the traditional theory about the role of ammonia-oxidizing bacteria (AOB) and archaea as catalysts in the first step of nitrification no longer holds (Van Kessel et al., 2015; Yu et al., 2018; Osburn and Barrett, 2020). Moreover, the discovery of CAOB has complicated our understanding of nitrification, making it difficult to evaluate the roles of different nitrifiers, especially in soils with frequent N nutrient additions (Xia et al., 2018; Li et al., 2019; Xu et al., 2020). The comammox *Nitrospira* are indistinguishable from *Nitrospira* performing only nitrite oxidation by their ribosomal sequences, and the gene encoding ammonia monooxygenase subunit A (*amoA*) has been used to examine their phylogeny, abundance, and communities in environmental samples. The phylogeny of *amoA* divides CAOB into two distinct branches referred to as clades A and B. Clade A is more abundant in alkaline soil samples and nutrient-rich arable soil relative to clade B (Kits et al., 2017; Pjevac et al., 2017; Koch et al., 2019). CAOB has a high affinity for ammonia and is competitive in oligotrophic habitats. Studies by Hu and He (2017), Osburn and Barrett (2020), and Li et al. (2021) have documented that edaphic factors such as available nitrogen, pH, soil organic carbon, and carbon-to-nitrogen ratio influence the CAOB abundance, diversity, and composition in arable soils. Variations in soil conditions may have variable effects on nitrification communities and consequently impact their roles in N_2_O emissions (Li et al., 2019; Schmidt et al., 2019). Researchers successfully isolated a culture of *Nitrospira inopinata*, a comammox bacterium, from a microbiological biofilm. This bacterium exhibited a strong affinity for ammonia, providing it with a competitive edge in environments with low nutrient levels. The comammox cultures, such as Ca. *Nitrospira nitrosa* and Ca. *Nitrospira nitrificans*, have been enriched from man-made systems and belong to clade A. The diversity of AOB and CAOB nitrifiers and their response to environmental factors and N fertilizer in soil ecosystems are essential in agricultural systems (Daims et al., 2015; Van Kessel et al., 2015; Koch et al., 2019). However, the role of AOB and CAOB communities within the semi-arid environs remains limited, hence the need for further research.

Maize is a widely cultivated crop in regions worldwide, including semi-arid areas such as the Loess Plateau. The semi-arid Loess Plateau (SALP), located in the northwestern region of China, is recognized as a highly vulnerable global agroecosystem due to its dependence on limited and unpredictable precipitation. To address this challenge, farmers often employ the plastic mulch technique to enhance maize yield. This technique elevates soil humidity and temperature and increases fertilizer usage. The increase in fertilizer usage enhances N_2_O emissions within the maize field (Bu et al., 2014; Zhang F. et al., 2018; Govindasamy et al., 2023). The use of plastic mulch can reduce water evaporation and retain moisture to meet the needs of crops in their crucial growth stages, thereby increasing crop yield and water use efficiency (Lamptey et al., 2019; Fudjoe et al., 2021).

In the rhizosphere, microorganisms and plant roots interact, leading to enhanced microbial nitrogen conversion rates facilitated by the release of oxygen from the roots (Zhang B. et al., 2018; Lin et al., 2020). Understanding the collaborations between different taxa of AOB and CAOB is crucial for evaluating soil functions. Co-occurrence networks can help reveal their taxa's ecological and microbe-to-microbe associations. Additionally, these networks are capable of handling large datasets (Zhang B. et al., 2018; Fudjoe et al., 2021). However, it is essential to identify keystone taxa that maintain function and structure within soil microbial communities (Williams et al., 2014; Mamet et al., 2019). Despite ongoing research efforts, there is still limited knowledge regarding the co-occurrence of the canonical AOB and CAOB genes in the semi-arid Loess Plateau area. Existing studies have not sufficiently addressed this gap, and there remains a lack of understanding of how agricultural management practices influence these microbial communities. Hence, a detailed understanding is crucial. Considering this, this study aimed to: (1) examine how the diversity, and composition of AOB and CAOB communities are influenced by long-term inorganic and organic fertilization; (2) assess the impact of inorganic and organic fertilizer on N_2_O emissions, maize yield, and NUE; and (3) explore the niche differentiation and co-occurrence networks between AOB and CAOB communities regarding N_2_O emissions, maize yield, and NUE. We hypothesized that AOB exhibited higher amoA gene transcriptional abundances and nitrification affinity activities under fertilization regimes (i.e., urea and organic nitrogen fertilizers) than CAOB due to their copiotrophic lifestyle and functional patterns.

## Materials and methods

### Field experimental description

The field study was conducted at the Rainfed Agricultural Experimental Station of Gansu Agricultural University, located in Gansu Province, NW China (35°28′N, 104°44′E, elevation: 1,971 m above sea level). The climate in this Loess Plateau area is semi-arid with 140 frost-free days annually. The region has steep terrain prone to erosion. The region's soil is aeolian, referred to as Huangmian, and has a sandy, loamy texture with at least 50% sand content. It is classified as Calcaric Cambisol according to the Food and Agriculture Organization (FAO) (1990) soil classification system. The other physiochemical properties of the soil measured before starting the experiment are shown in Supplementary Table S1. The average annual temperature and precipitation at the study site are recorded as 10.8°C and 400 mm, respectively. The yearly evaporation rate was 1,531 mm, and the average annual radiation was 5930 MJ m^−2^. Between January and August, the temperature fluctuated between −23 and 37°C, and the average annual precipitation was 390.8 mm and 369.2 mm during the 2020 and 2021 cropping seasons.

The experiment followed a randomized complete block design with five treatments and three replicates per treatment. The five treatments were as follows: (i) no fertilizer (NA); (ii) inorganic fertilizer (CF) contained 200 kg N ha^−1^ of urea and 150 kg P_2_O_5_ ha^−1^ of triple superphosphate; (iii) inorganic fertilizer plus organic fertilizer (SC) contained 3.03 t ha^−1^ of organic fertilizer, 100 kg N ha^−1^ of urea, and 120 kg P_2_O_5_ ha^−1^ of triple superphosphate; (iv) organic fertilizer (SM) contained 6.06 t ha^−1^ of organic fertilizer and 90 kg P_2_O_5_ ha^−1^ of triple superphosphate; and (v) maize straw (MS) contained 28.5 t ha^−1^, 100 kg N ha^−1^ of urea, and 36 kg P_2_O_5_ ha^−1^ of triple superphosphate. There were 15 plots, each measuring 3 m × 14.2 m. Treatments SC and MS were applied at the same N input rate. Urea, commercial organic fertilizer, and maize straw were broadcast and integrated into the top 20 cm soil layer.

The experiment was initiated in 2012, but this study reports data from the 2020 and 2021 cropping seasons. Before planting, the soil in the plots was manually inverted with shovels to a depth of 20 cm. Wide (0.7 m) and narrow (0.4 m) ridges were covered with colorless plastic film, and holes were made in the film over the furrows to collect precipitation. After placing the film over the soil, it was perforated using a handheld device (Lamptey et al., 2019). Maize seeds of the Pioneer 335 cultivar were sown in furrows (Supplementary Figure S1) at a density of 52,500 plants ha^−1^ in late April. Glyphosate (30%) was sprayed before planting to control weeds, and manual weeding was carried out when necessary, during the season. The maize grain was harvested in late September.

### Soil sampling and property analysis

During the silking stage of the 2020 and 2021 cropping seasons, soil samples were gathered from the maize fields of the experiment. A total of 15 soil samples were randomly collected, with three replicates for each of the five treatments. The soil samples were specifically taken from the rhizosphere, which refers to the soil closely adhering to the root crowns, where the dense root system ensured that all the soil was influenced by the roots. The soil samples collected from each plot were sieved through a 2-mm sieve to eliminate any residues and then combined to create a collective sample. Immediately after collection, the soil samples were preserved on dry ice, transported to the laboratory, and preserved at −80°C for molecular analysis. A portion of the remaining subsample was kept at 4°C for microbial biomass C and N analyses, while the other soil samples were air-dried to facilitate subsequent chemical analysis.

To measure the soil pH, a deionized soil suspension was prepared with a soil-to-water ratio of 1:2.5 (mass to volume). A pH meter (Mettler Toledo FE20, Shanghai, China) was used for the pH measurement. Soil organic carbon (SOC) was determined using a modified Walkley-Black wet oxidation method, while total nitrogen (TN) was analyzed using the Kjeldahl method (Bao, 2000). Soil nitrate–nitrogen (${\text{NO}}_{3}^{-}$-N) and ammonium–nitrogen (${\text{NH}}_{4}^{+}$-N) contents were extracted with 2 M KCl and determined using a flow injection auto-analyzer (FLA Star 5000 analyzers, Foss, Denmark) (Bremner, 1965). Soil dissolved organic N (DON) in H_2_O (1:1) was analyzed using the Multi N/C^®^2100 Analyzer (ANALYTIKJENA, Germany) (Ghani et al., 2003). Available phosphorus (AP) was measured using the molybdenum-blue method after extraction with sodium bicarbonate (Olsen et al., 1954). Soil water content (SWC) was measured by oven-drying the soil at 105°C for 24 h (Fudjoe et al., 2021).

### Measurement of potential nitrification activity, maize productivity, and nitrogen use efficiency

To measure potential nitrification activity (PNA), the chlorate inhibition method was employed. In brief, 5 g of soil was weighed and placed in a 50-ml centrifuge tube, along with 20 ml of phosphate buffer solution (PBS) containing specific concentrations of NaCl (8.0 g/L), KCl (0.2 g/L), Na_2_HPO_4_ (1.44 g/L), and NaH_2_PO_4_ (0.2 g/L). Ammonium sulfate (NH_4_)_2_SO_4_ was added to 1 mM concentration, and potassium chlorate (KClO_3_) was added to inhibit nitrite oxidation (final concentration = 10 mM). The mixture was then incubated in the dark at a temperature of 25°C with shaking slurry at 180 r min^−1^ for 24 h. Following incubation, the inhibited nitrite was extracted by adding 5 ml of 2 M KCl solution, and its concentration was measured using a spectrophotometer at a wavelength of 540 nm with N-(1-naphthyl) ethylenediamine dihydrochloride (Kurola et al., 2005; Ullah et al., 2020).

The aboveground biomass and grain yield for the maize cropping seasons of 2020 and 2021 were determined by oven-drying at 105°C for 45 min and subsequently drying to constant weight at 85°C. Grain and forage yields (kg ha^−1^) were extrapolated (Alhassan et al., 2018). The NUE was calculated by subtracting the nitrogen uptake in the treatment without nitrogen fertilizer from the nitrogen buildup in the treatment with nitrogen fertilizer. This difference was then divided by the nitrogen application rate (Cardenas et al., 2019).

### Collection and determination of N_2_O emission samples

The gas samples were obtained using the static chamber technique, and the concentration of nitrous oxide (N_2_O) was measured using gas chromatography (Agilent 7080B, Santa Clara, USA) at monthly or bi-monthly intervals throughout the maize cropping seasons of 2020 and 2021. Each sealed container (0.38 m × 0.35 m × 0.36 m) was constructed with an opaque outer lid covered with crenelated container foil to minimize the impact of thermal heat during gas sampling. Furthermore, two fans were installed inside the lid to ensure proper gas circulation prior to sampling. To minimize the impact of diurnal temperature variations, N_2_O gas samples were collected using a 60-ml plastic gas-tight syringe within a specific time frame (within 9:00 and 11:00) during different sampling periods (0, 10, and 20 min after chamber closure). The collected gas samples were then stored in airtight aluminum bags (Dalian Delin Gas Packing, China). Gas chromatography (Agilent 7890A, United States) with an electron capture detector was utilized to analyze the gas samples.

1. The N_2_O fluxes (NF, mg m^−2^ h^−1^) were calculated using Eq. (1) based on the procedure detailed by Huang et al. (2019);
(1) $$\begin{array}{l} NF=\frac{273}{273+T}\times \frac{44}{22.4}\times 60\times 10^{-3}\times h\times \;\frac{dc}{dt} \end{array}$$
  In the equation, *T* (°C) represents the air temperature, 44 denotes the molecular weight of N_2_O, 22.4 (L mol^−1^) corresponds to the molecular volume at 101 kPa, 60 × 10^−3^ is a conversion factor, *h* represents the height of the chamber, and dc/dt represents the rate of change in N_2_O concentration (c) per unit of time (t).
2. N_2_O cumulative emissions (NE, Kg ha^−1^) were calculated using Eq. (2) based on Tao et al. (2018);
(2) $$\begin{array}{l} NE=\sum \left[\frac{NF_{i+1}+NF_{i}}{2}\times \left(t_{i+1}-t_{i}\right)\times 24\times 10^{-2}\right] \end{array}$$
  where *i* + 1 and *i* are the last and current measurement dates, respectively, and *t* is the number of days after sowing.

### Quantitative polymerase chain reaction of functional gene communities

The total genomic DNA was obtained from the rhizosphere soil (0.5 g dry weight) using the DNA Isolation Kit (MoBio, Carlsbad, CA, USA). Following the extraction, a Wizard DNA Clean-Up System (Axygen Bio, USA) was utilized to purify the extracted DNA. The DNA samples were then kept at −80°C until they were analyzed. The copy numbers of the amoA–AOB and comammox *Nitrospira* genes were determined using quantitative polymerase chain reaction (qPCR) with the specific primer set described in Supplementary Table S2. For qPCR, the 20 μl reaction mixture consisted of 7.2 μl of aseptic water, 0.4 μl of each primer (10 mM), 10 μl of GoTaq^®^ qPCR Master Mix (Promega, USA), and 2 μl of template DNA. A calibration series (ranging from 10^2^ to 10^8^ copies) of plasmid DNA was employed to construct standard curves for quantifying the copy numbers of target genes (AOB and CAOB). All qPCR analyses were initiated with an initial denaturation stage at 95°C for 3 min, followed by 40 cycles (with plate-reading) consisting of 30 s at 95°C and 45 s at 60°C, subsequently concluding with a final melt curve step spanning 72 to 95°C. The qPCR process was performed three times, and high amplification efficiencies (>97%) were achieved, supported by standard curve *r*^2^ values exceeding 0.99%.

### High-throughput sequencing of functional gene amplicons

DNA sequencing was employed to investigate the relative abundance and composition of the *amoA*–AOB and comammox *Nitrospira* genes. For the forward primers, a 7-bp unique barcode sequence was appended, and the concentration of the refined PCR products was quantified using a TBS-380 fluorometer (Turner Biosystems, CA, United States). The PCR products were then diluted and subjected to paired-end sequencing on an Illumina MiSeq sequencer (Shanghai Personal Biotechnology, Co., Ltd., Shanghai, China). Detailed information regarding the primer pairs, reaction mixtures, and thermal cycling conditions employed to amplify fragments of all genes is shown in Supplementary Table S2. After the amplification step, the PCR products from the genes were retrieved from agarose gels and subjected to purification using a universal DNA Purification Kit (Tiangen Co., Beijing, China). To identify low-quality sequences, the raw sequences were screened for quality using Quantitative Insights Into Microbial Ecology (QIIME) (Caporaso et al., 2010). The Usearch tool was employed to screen for chimeric assembled sequences, while the FrameBot tool from the Ribosomal Database Project (RDP) was used (Edgar, 2013). The FunGene Pipeline was utilized to exclude sequences of low quality (Edgar and Flyvbjerg, 2015). Operational taxonomic units (OTUs) were defined using the CD-HIT approach within MOTHUR, with a 3% difference threshold in nucleotide sequences (Schloss et al., 2009). Homologs and the closest sequences in GenBank were identified using the MEGA software. Moreover, representative sequences from each OTU were aligned with the most closely related sequences and additional reference sequences that were retrieved using the MEGA software, as described by Tamura et al. (2013). The *amoA–*AOB and comammox *Nitrospira* gene sequences were deposited in the NCBI Sequence Read Archive (SRA) database under specific accession numbers, namely PRJNA722852 and PRJNA967803. A neighbor-joining tree was constructed in MEGA 6 using a Kimura 2-parameter distance and 1000 bootstrap replicates to classify *amoA*–AOB and comammox *Nitrospira* OTUs, following the nomenclature described by Wang et al. (2017) and Li et al. (2021), respectively.

### Statistical analysis

Data analysis was performed using SPSS version 22 (IBM Corporation, Chicago, USA, 2013). A one-way analysis of variance (ANOVA) was utilized to examine the treatment means of the copy number of *amoA–*AOB and comammox *Nitrospira* genes, soil PNA, grain yield, and aboveground biomass. Duncan's multiple range tests (DMRTs) at a 95% confidence level (*p* ≤ 0.05) were used to differentiate between the means. The alpha diversity indices (Shannon index, Simpson index, and Chao1 richness) of the functional genes were carried out using R software (version 3.5.3). The correlation analyses were performed to assess the connections between grain and biomass yield, soil water content, and soil chemical properties. Redundancy analysis (RDA) was implemented through the “vegan” package in R to utilize the impact of soil physiochemical properties on functional genes. Principal component analysis (PCA) was conducted using R statistical software to examine the variations among fertilization treatments.

Co-occurrence networks were employed to classify significant associations among taxa in the AOB and CAOB communities. A total of 15 soil fertilization treatment samples (consisting of three replications for each of the five treatments) were combined for analysis. The operational taxonomic units (OTUs) present in all treatment replicates were selected for network analysis. Pearson's correlation, Bray–Curtis, and Kullback–Leibler dissimilarities were employed in a collaborative approach. A true co-occurrence network was defined as a statistically significant association between species, indicated by a correlation coefficient (*r*) >0.8 or < -0.8 and a *p*-value of 0.01. To assess the reliability of the connections, permutation and bootstrap distributions were computed with 1,000 iterations. The network was visualized using the Fruchterman–Reingold algorithm in Gephi (version 0.9.2). Various topological properties of the network were calculated, such as the number of nodes and edges, average clustering coefficient, average degree, average path length, closeness centrality, network centrality, and modularity. Potential keystone taxa were identified as OTUs with higher degrees of centrality using the methodology outlined by Berry and Widder (2014).

To explore important predictors of the AOB and CAOB communities, maize productivity, and N_2_O emissions, a random forest modeling approach was utilized. The analysis incorporated soil variables, and the forest package developed by Liaw and Wiener (2002) was utilized. The importance of analysis in the model was computed using the “A3R” package by Fortmannroe (2015), and the statistical significance of each forecaster was assessed using the “rfPermute” package developed by Archer (2020). The major forecasters obtained from the random forest analysis were utilized to investigate the direct and indirect effects of soil properties on abiotic and biotic variables, including physiochemical soil properties, biomass, network modules, soil AOB and CAOB communities, maize productivity, PNA, and N_2_O. The analysis was performed using AMOS 21.0 in SPSS (SPSS, Inc., Chicago, IL). Before modeling, the normality of the data distribution was assessed. The structural equation model (SEM) was applied, and the model fitness was evaluated using the chi-square test (χ^2^, *p* > 0.05), root mean square error of approximation (RMSEA), and goodness-of-fit index (GFI) following the methodology described by Sahoo (2019).

## Results

### Soil properties, maize productivity, and nitrogen use efficiency

The analysis of variance indicated significant differences among treatments for most soil indices (TN, ${\text{NO}}_{3}^{-}$-N, AP, SOC, DON, and SWC) in the 2020 and 2021 cropping seasons, except for ${\text{NH}}_{4}^{+}$-N. The soil pH varied significantly across treatments, ranging from 8.10 to 8.80. However, no fertilizer (NA) treatment had a higher pH than the fertilization treatments (MS, SM, SC, and CF; Table 1). The SOC, ${\text{NO}}_{3}^{-}$-N, AP, and DON tended to be higher in 2021 cropping season than in the 2020 cropping season, and they increased across fertilization treatments. Specifically, the MS, SM, and SC treatments increased SOC by 17.3%, 15.4%, and 15.1%, and increased DON by 69.7%, 63.3%, and 9.2% compared to NA, respectively. However, CF and MS significantly increased TN, while SM and SC treatments were high in AP compared to NA treatments in 2020 and 2021 cropping seasons. SWC exhibited a significant increase in SC, SM, and MS treatments compared to NA. There were significant differences (*p* < 0.05) observed in the ${\text{NO}}_{3}^{-}$-N concentrations during the 2020 and 2021 cropping seasons in the CF treatment relative to the SC, SM, MS, and NA treatments (Table 1).

**Table 1:** Soil chemical characteristics under different fertilization treatments in the rhizosphere soil.

**Year**	**Indices**	**NA**	**CF**	**SC**	**SM**	**MS**
2020	pH	8.80a	8.43b	8.37b	8.19c	8.68ab
	TN (g kg^−1^)	0.85b	0.93a	0.94a	0.98a	0.99a
	SOC (g kg^−1^)	7.48c	7.93c	8.81b	8.84b	9.81a
	${\text{NO}}_{3}^{-}$-N (mg kg^−1^)	17.84c	30.81a	28.40ab	25.40b	21.93bc
	${\text{NH}}_{4}^{+}$-N (mg kg^−1^)	15.33a	16.07a	14.87a	15.81a	16.53a
	AP (mg kg^−1^)	9.73c	16.70ab	18.32ab	19.81a	15.14b
	DON (mg kg^−1^)	10.89b	12.42b	11.89b	17.78a	18.48a
	SWC (%)	23.11b	28.21b	32.42a	31.93a	28.63b
2021	pH	8.66a	8.32c	8.44bc	8.45bc	8.54b
	TN (g kg^−1^)	0.80c	1.15ab	1.03b	1.07b	1.22a
	SOC (g kg^−1^)	7.84c	8.94bc	9.78b	9.96b	11.77a
	${\text{NO}}_{3}^{-}$-N (mg kg^−1^)	18.73c	32.48a	29.29b	26.08b	23.76bc
	${\text{NH}}_{4}^{+}$-N (mg kg^−1^)	16.61a	17.82a	15.52a	17.08a	18.92a
	AP (mg kg^−1^)	10.69c	17.74b	20.77a	19.16ab	16.81bc
	DON (mg kg^−1^)	11.49c	19.91a	14.41b	17.97ab	12.55b
	SWC (%)	13.34c	23.39a	22.53ab	15.39bc	17.37b

Values are expressed as mean with letters indicating significant differences based on Duncan's HSD test (p < 0.05).

TN, total nitrogen; SOC, soil organic carbon; ${\text{NO}}_{3}^{-}$-N, nitrate nitrogen; ${\text{NH}}_{4}^{+}$-N, ammonia nitrogen; AP, available phosphorus; DON, dissolved organic nitrogen; SWC, soil water content (0–20 cm); NA, no fertilization; CF, mineral fertilizer; SC, mineral fertilizer plus commercial organic fertilizer; SM, commercial organic fertilizer; MS, maize straw.

Fertilization treatments significantly (*p* < 0.05) enhanced maize productivity relative to no fertilizer (NA) treatment in the 2020 and 2021 cropping seasons (Supplementary Table S3). Grain yield during the 2020 and 2021 cropping seasons under CF and SC treatments increased by (61.99%, 58.24%) and (63.99%, 61.15%), respectively, compared to NA treatment. The CF treatment yielded the highest aboveground biomass, compared to the SC, MS, and SM treatments. In 2020, the CF, SC, MS, and SM treatments increased aboveground biomass by 2.8, 2.6, 1.7, and 1.4 times, respectively (Supplementary Table S3). In 2021, these treatments resulted in 2.2, 2.1, 1.4, and 1.3 times higher aboveground biomass. The NUE exhibited a similar pattern as maize productivity. In 2020, the CF, SC, and SM treatments enhanced NUE by 60%, 56%, and 24%, respectively compared to the MS treatment. In 2021, these treatments increased NUE by 56%, 53%, and 22%, respectively compared to the MS treatment (Supplementary Table S3).

### Potential nitrification activity and N_2_O emissions

**Figure 1:**
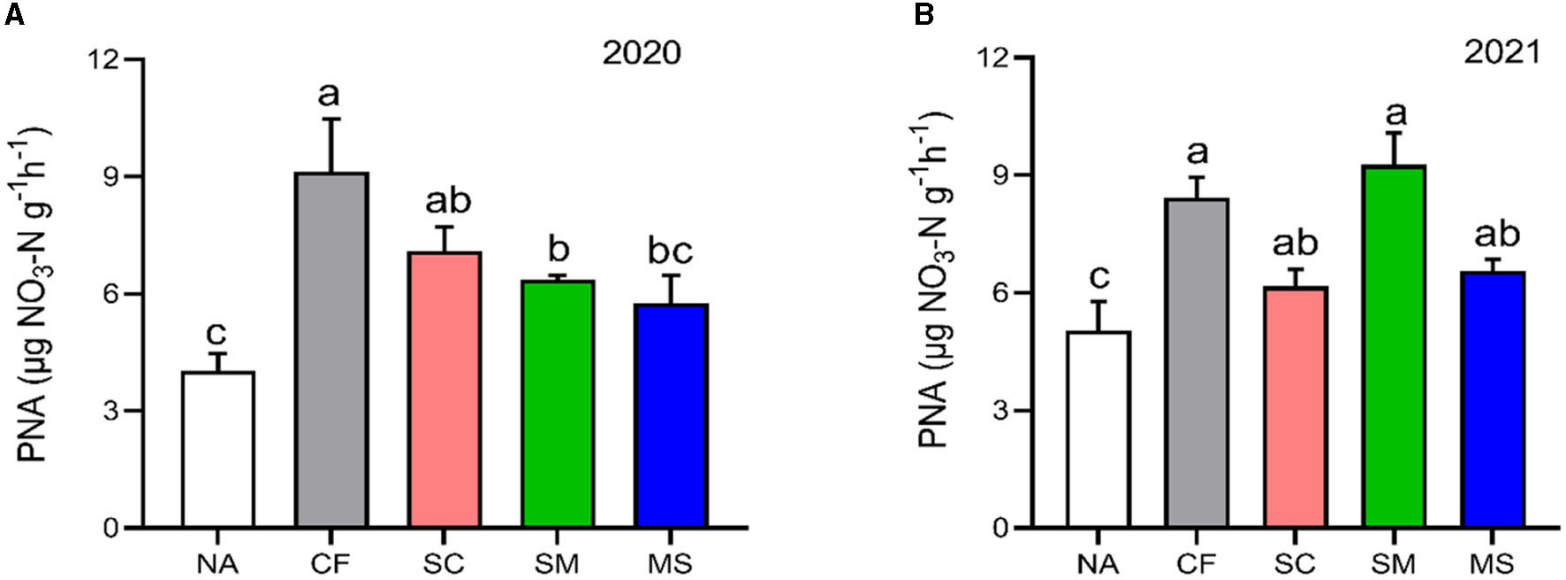
Potential nitrification activity (PNA) under different soil fertilization treatments in the **(A)** 2020 and **(B)** 2021 cropping seasons. Bars (*n* = 3) with different lowercase letters specify significant differences based on Duncan's HSD test (*p* < 0.05). NA, no fertilization; CF, inorganic fertilizer; SC, inorganic plus organic fertilizer; SM, organic fertilizer; MS, maize straw.

The PNA index in the 2020 and 20221 cropping seasons increased significantly (*p* < 0.05) under CF (55.84%, 40.32%), SC (43.19%, 18.39%), SM (36.66%, 45.68%), and MS (30.07%, 23.36%) treatments relative to NA treatment, respectively (Figure 1).

**Figure 2:**
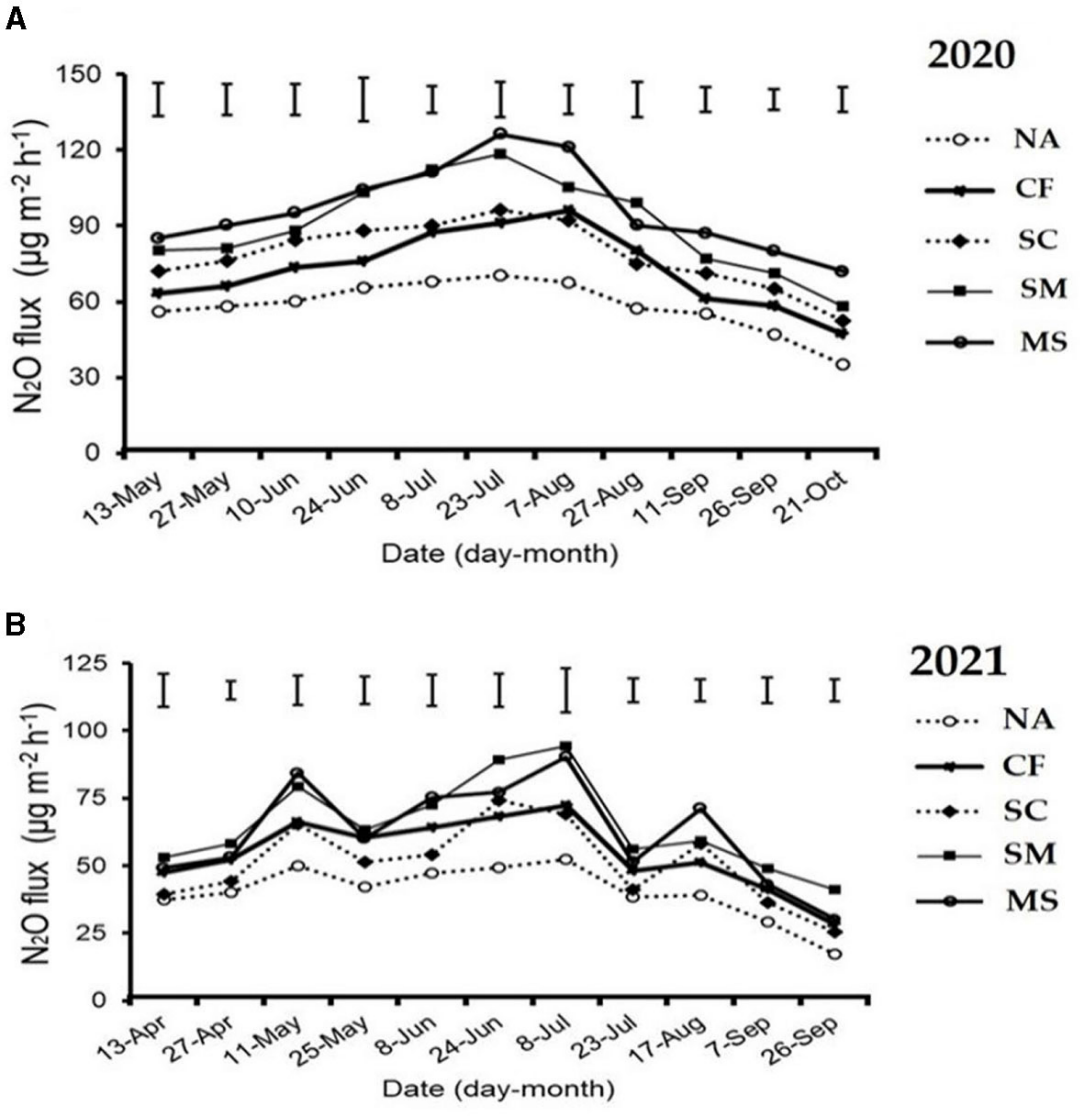
Seasonal variations of N_2_O flux emissions; **(A)** in 2020 and **(B)** in 2021 as influenced by fertilization treatments. The vertical bars represent the least significant difference (LSD) at a *p*-value of < 0.05. Bars (*n* = 3). NA, no fertilization; CF, inorganic fertilizer; SC, inorganic plus organic fertilizer; SM, organic fertilizer; MS, maize straw.

**Figure 3:**
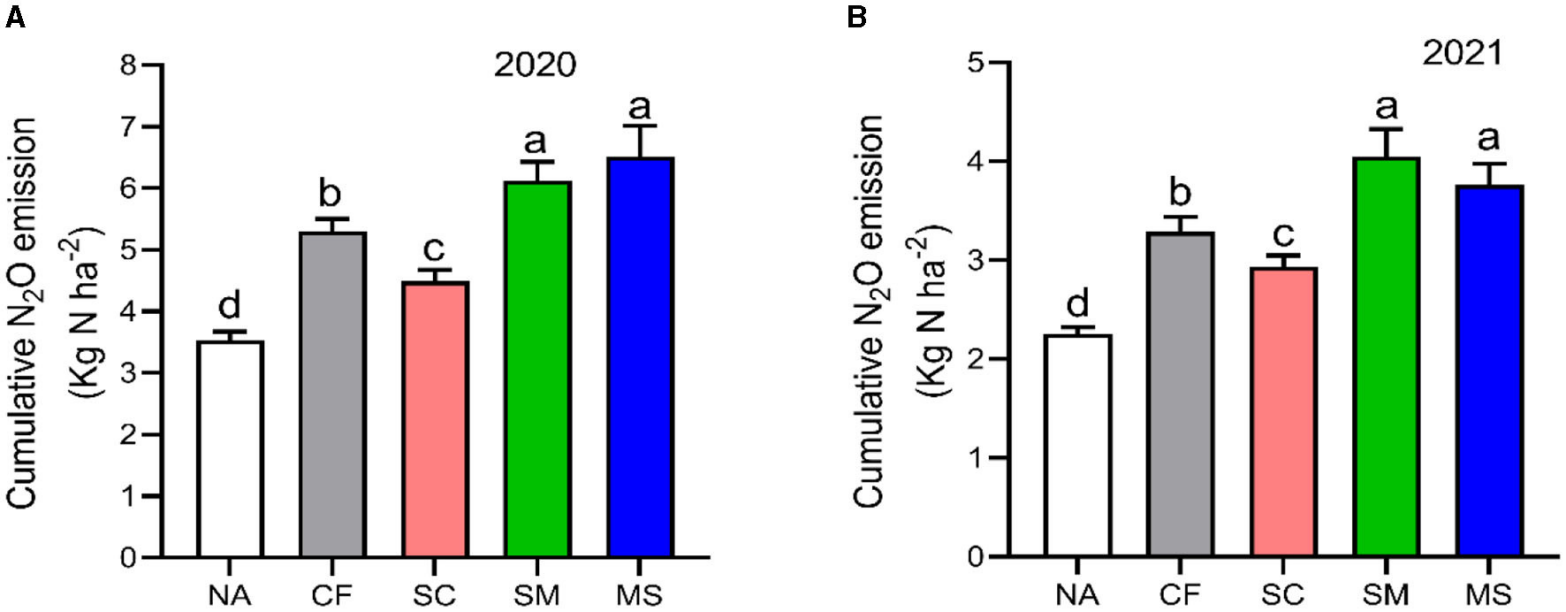
Seasonal variations of N_2_O cumulative emissions; **(A)** in 2020 and **(B)** in 2021 as influenced by fertilization treatments. The vertical bars represent the least significant difference (LSD) at a *p*-value of < 0.05. Bars (*n* = 3) with different lowercase letters indicate significant differences based on Duncan's HSD test (*p* < 0.05). NA, no fertilization; CF, inorganic fertilizer; SC, inorganic plus organic fertilizer; SM, organic fertilizer; MS, maize straw.

The maximum peaks observed in N_2_O flux emissions occurred in July, while the minimum levels were recorded in October and September through all fertilization treatments in the 2020 and 2021 cropping seasons of this study (Figure 2A). Furthermore, the release of N_2_O flux emissions was significantly (*p* < 0.05) higher in the SM, MS, and CF treatments relative to SC and NA treatments in the growing seasons (Figure 2A). The highest N_2_O emission flux in the 2020 and 2021 cropping seasons was observed under SM, followed by MS treatment at (115.5 and 110 mg m^−2^ h^−1^) and (98 and 80 mg m^−2^ h^−1^), respectively, while the lowest was under NA treatment at 25 and 18 mg m^−2^ h^−1^, respectively (Figures 2A, B). The cumulative N_2_O emissions in 2020 cropping season were 45.7% greater with MS treatment relative to NA, while in the 2021 cropping season, they increased significantly under CF, SC, SM, and MS treatments at 31.5%, 23.3%, 44.3%, and 40.1%, respectively compared to NA treatment and were ranked as SM > MS > CF > SC (*p* < 0.05; Figures 3A, B). The SC treatment indicated a lower emissions rate and cumulative N_2_O emissions than the CF treatment (Figures 3A, B).

### Community structure of AOB and CAOB

**Figure 4:**
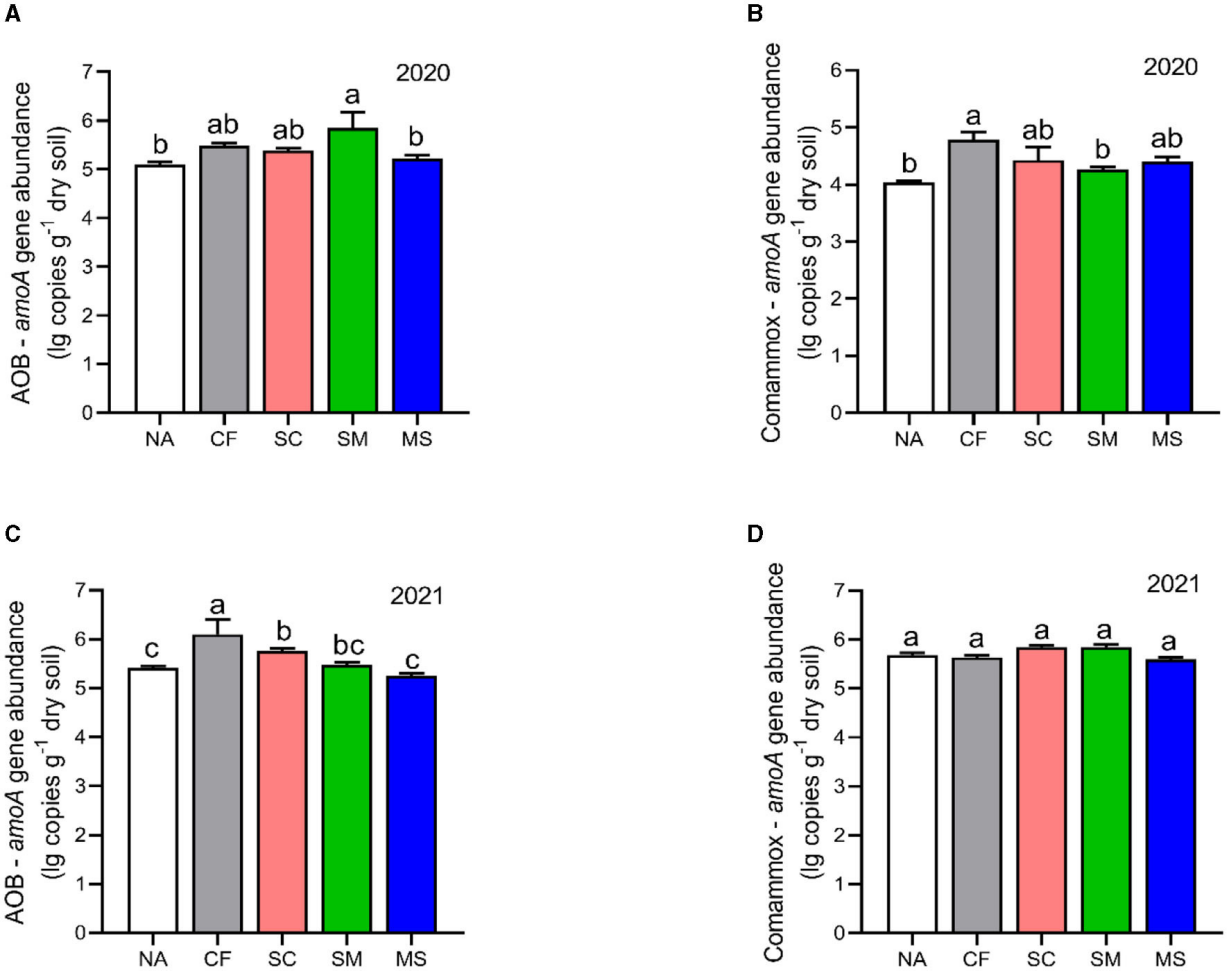
Gene copy numbers of **(A–C)** *amoA*-AOB and **(B–D)** comammox *Nitrospira* (CAOB) genes as influenced by fertilization treatments. Values are mean ± standard error (*n* = 3), with different lowercase letters indicating significant differences based on Duncan's HSD test (*p* < 0.05). NA, No fertilization; CF, inorganic fertilizer; SC, inorganic plus organic fertilizer; SM, organic fertilizer; MS, maize straw.

The copy numbers of AOB were higher than CAOB across the 2020 and 2021 cropping seasons. The AOB abundance under SM, CF, SC, and MS treatments increased significantly by 12.77%, 6.9%, 5.36%, and 2.23%, respectively compared with the NA in the 2020 cropping season (Figure 4A). In the 2021 cropping season, the abundance of AOB was considerably greater (*p* < 0.05) under the CF, SC, and SM treatments compared to the NA and MS treatments (Figure 4C). Compared to the NA treatment, CF, SC, MS, and SM increased significantly by 15.48%, 8.65%, 8.03%, and 5.01%, respectively, under the CAOB abundance (*p* < 0.05, Figure 4B) in the 2020 cropping season, whereas in 2021, the growing season had no significant effect among the treatments (*p* > 0.05, Figure 4D).

The diversity index of AOB and CAOB OTUs was significantly higher under SM and SC treatments relative to NA treatment (Table 2). The CF and SM treatments were significantly higher in the diversity indices of AOB of Chao1 richness and Shannon index (*p* < 0.05) compared to the NA treatment (Table 2). Similarly, for the comammox *Nitrospira* region, the Chao1 richness and Shannon indices under CF, SC, SM, and MS increased by 1.18-, 1.01-, 1.16-, and 1.17-fold and by 1.03-, 1.05-, 1.06-, and 1.04-fold relative to NA treatments (Table 2).

**Table 2:** Alpha diversity indices of *amoA*–AOB and comammox *Nitrospira* (CAOB) at the similarity level of 97% under the influence of fertilization treatments in the rhizosphere soil.

**Nitrifiers**	**Treatments**	**OTUs**	**Chao1**	**Shannon**
AOB nitrifiers	NA	1,796.3 ± 85.2^c^	2,143 ± 56.6^c^	5.1 ± 0.24^c^
	CF	2,472.6 ± 47.1^ab^	3,442 ± 14.3^a^	5.75 ± 0.16^ab^
	SC	1,859 ± 55.6^bc^	2,177 ± 9.8^bc^	5.73 ± 0.29^ab^
	SM	2,604 ± 94.3^a^	2,991 ± 29.6^ab^	5.47 ± 0.38^abc^
	MS	2,062 ± 76.5^b^	2,429 ± 20.5^b^	6.14 ± 0.15^a^
	*p*-value	< 0.012	0.001	0.004
CAOB nitrifiers	NA	1,145 ± 63.1^c^	1,440 ± 44.5^b^	6.50 ± 0.18^c^
	CF	1,410 ± 47.1^b^	1,701 ± 11.3^a^	6.75 ± 0.19^b^
	SC	1,953 ± 55.6^a^	1,468 ± 29.6^b^	6.81 ± 0.21^ab^
	SM	1,208 ± 83.1^bc^	1,667 ± 18.4^a^	6.88 ± 0.38^a^
	MS	1,839 ± 65.5^a^	1,697 ± 23.6^a^	6.78 ± 0.17^b^
	*p*-value	< 0.002	0.001	0.036

The data include the mean with standard error. Values with the same alphabet are not statistically different, but values with different letters indicate a statistically significant difference between fertilization treatments at Duncan's HSD test (p < 0.05).

NA, no fertilization; CF, inorganic fertilizer; SC, inorganic plus organic fertilizer; SM, organic fertilizer; MS, maize straw.

**Figure 5:**
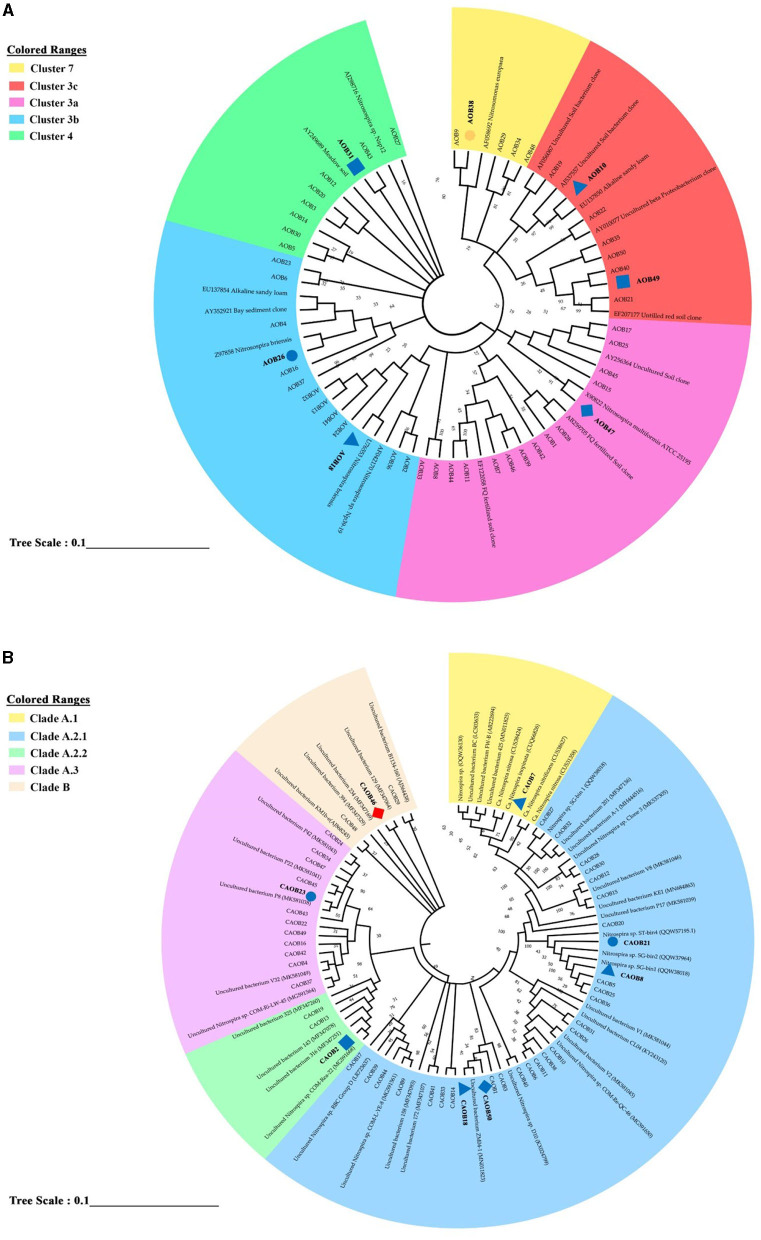
Phylogenetic neighbor-joining tree based on the genus level of **(A)** *amoA*–AOB genes and **(B)** comammox *Nitrospira* (CAOB) genes. The study provided information on the top 50 most prevalent sample sequences and their closest matches in the custom FunGene amoA sequence database. Additionally, the NCBI taxonomic classification of the database entries was included. The results also show the percentage of clone trees in which related taxa were grouped in the bootstrap test. A bootstrap value exceeding 50% is indicated next to the branches. Different shapes and colors were used to represent significant differences between the study's various fertilizer treatments and no fertilization. A circle represented a significant difference between inorganic fertilizer (CF) and no fertilization (NA). In contrast, a triangle shape represented a significant difference between inorganic plus organic fertilizer (SC) and no fertilization (NA). A rectangle shape was used to represent a significant difference between organic fertilizer (SM) and no fertilization (NA), and a diamond shape represented a significant difference between maize straw only (MS) and no fertilization (NA). Blue indicated a significantly higher abundance than the control treatment, while red indicated a significantly lower abundance than no fertilization (NA).

Using the 50 most prevalent OTUs across all treatments, a phylogenetic tree was created to examine the community composition of the AOB and CAOB populations (Figures 5A, B). Among the AOB community, 50 dominant OTUs are seen in Figure 5A. Five clustered lineages of *Nitrosospira*: 7, 3c, 3a, 3b, and 4 were identified. Fertilization significantly increased across seven AOB OTUs (ANOVA, *p* < 0.05; Figure 5A and Supplementary Figure S2A). AOB38, AOB10, AOB49, and AOB47 were significantly higher in CF, SC, MS, and SM than the no fertilizer treatments (NA) affiliated with *Nitrosospira* Cluster 7, 3c, and 3a, respectively (Figure 4A and Supplementary Figure S2A). AOB7 was significantly higher in MS relative to other N fertilization treatments and NA (Figure 5A and Supplementary Figure S2A). In *Nitrosospira* 3b, both AOB18 and AOB26 were associated with *Nitrosospira briensis*, which was significantly higher in SC and CF than in the NA treatment (Figure 5A and Supplementary Figure S2A). The fertilization materials affected eight of the most abundant OTUs in the CAOB (ANOVA, *p* < 0.05). In the *Nitrospira* clade A.1, CAOB7 was associated with Ca. *N. inopinata*, Ca. *N. nitrosa*, and Ca. *N. nitrificans* and was significantly higher in SC than in NA treatment (Figure 5B and Supplementary Figure S2B). The primary descriptive sequences of the CAOB OTU were associated with the *Nitrospira* clade A.2.1 lineage (Figure 5B). CAOB21 and CAOB8 were high under organic and inorganic fertilizer materials (CF and SC) and no fertilizer treatment (NA), (which was related to *Nitrospira* sp. SG). At the same time, CAOB18 was significantly higher in SC and SM compared to NA treatment, respectively (Figure 5B and Supplementary Figure S2B). CAOB2 and CAOB23 were closely related to uncultured *Nitrospira* bacterium within *Nitrospira* Clade A.2.2 and A.3, and their contents were substantially greater in MS and CF compared to the NA treatments. CAOB46 was also greater in the NA group relative to the N fertilizer treatments and was affiliated with *Nitrospira* Clade B (Figure 5B and Supplementary Figure S2B).

Furthermore, the relative abundance of AOB by real-time PCR showed positive correlations to SOC (*r* = 0.51, *p* < 0.05), ${\text{NO}}_{3}^{-}$-N (*r* = 0.75, *p* < 0.01), PNA (*r* = 0.79, *p* < 0.01), N_2_O emission (*r* = 0.62, *p* < 0.05), and yield (*r* = 0.54, *p* < 0.05), but was negatively associated with pH (*r* = 0.63, *p* < 0.05; Table 3). The CAOB abundance by real-time PCR indicated a negative correlation with pH (*r* = −0.67, *p* < 0.05), TN (*r* = −0.72, *p* < 0.01), and N_2_O emission (*r* = −0.52, *p* < 0.05), while being positively associated with AP (*r* = 0.55, *p* < 0.05), ${\text{NO}}_{3}^{-}$-N (*r* = 0.61, *p* < 0.05), SWC (*r* = 0.55, *p* < 0.05), PNA (*r* = 0.84, *p* < 0.01), and yield (*r* = 0.68, *p* < 0.05; Table 3). However, the AOB diversity showed OTU, Shannon, and Simpson indices correlated positively with pH (*r* = 0.63, *p* < 0.05), SOC (*r* = 0.52, *p* < 0.05), and N_2_O emission (*r* = 0.78, *p* < 0.05), respectively. The Chao and Shannon indices correlated negatively with PNA (*r* = −0.52, *p* < 0.05) and DON (*r* = −0.66, *p* < 0.05), respectively (Supplementary Figure S3). Among the comammox *Nitrospira* diversity, the Chao, Shannon, and Simpson indices correlated positively with pH (*r* = 0.61, *p* < 0.05), ${\text{NO}}_{3}^{-}$-N (*r* = 0.74, *p* < 0.05), and SWC (*r* = 0.52, *p* < 0.05), respectively. The Simpson index was negatively associated with yield (*r* = −0.57, *p* < 0.01; Supplementary Figure S3).

**Table 3:** Pearson's correlation between gene copies of the ammonia-oxidizing bacteria (AOB), and comammox *Nitrospira* (CAOB) abundance, soil properties, PNA, N_2_O, and yield.

	**pH**	**TN**	**SOC**	**AP**	**${\text{NO}}_{3}^{-}$-N**	**${\text{NH}}_{4}^{+}$-N**	**DON**	**SWC**	**PNA**	**N_2_O**	**Yield**
AOB	−0.63^*^	0.04	0.51^*^	0.44	0.75^**^	−0.19	−0.27	0.42	0.79^**^	0.62^*^	0.54^*^
CAOB	−0.67^*^	−0.72^*^	−0.37	0.55^*^	0.61^*^	0.32	−0.22	0.55^*^	0.84^**^	−0.52^*^	0.68^*^

pH; TN, total nitrogen; SOC, soil organic carbon; ${\text{NO}}_{3}^{-}$-N, nitrate nitrogen; ${\text{NH}}_{4}^{+}$-N, ammonia nitrogen; AP, available phosphorous; DON, dissolved organic nitrogen; SWC, soil water content; PNA, soil potential nitrification activity; N_2_O, cumulative nitrous oxide emission; yield, sum of grain and biomass.

* Significant correlations are indicated at a p-value of < 0.05.

** Suggesting significant correlations at a p-value of < 0.01.

### Co-occurrence networks and prediction analysis

Distinct topological characteristics were observed in the AOB and CAOB populations based on the treatment's relative abundance. The AOB network community exhibited variations through Module I (28 nodes with 206 edges), Module II (21 nodes with 107 edges), Module III (37 nodes with 186 edges), and Module IV (41 nodes with 98 edges; Supplementary Table S4). On the other hand, the CAOB network community was Module I (23 nodes with 73 edges), Module II (20 nodes with 52 edges), and Module III (53 nodes with 153 edges), respectively (Supplementary Table S4).

**Figure 6:**
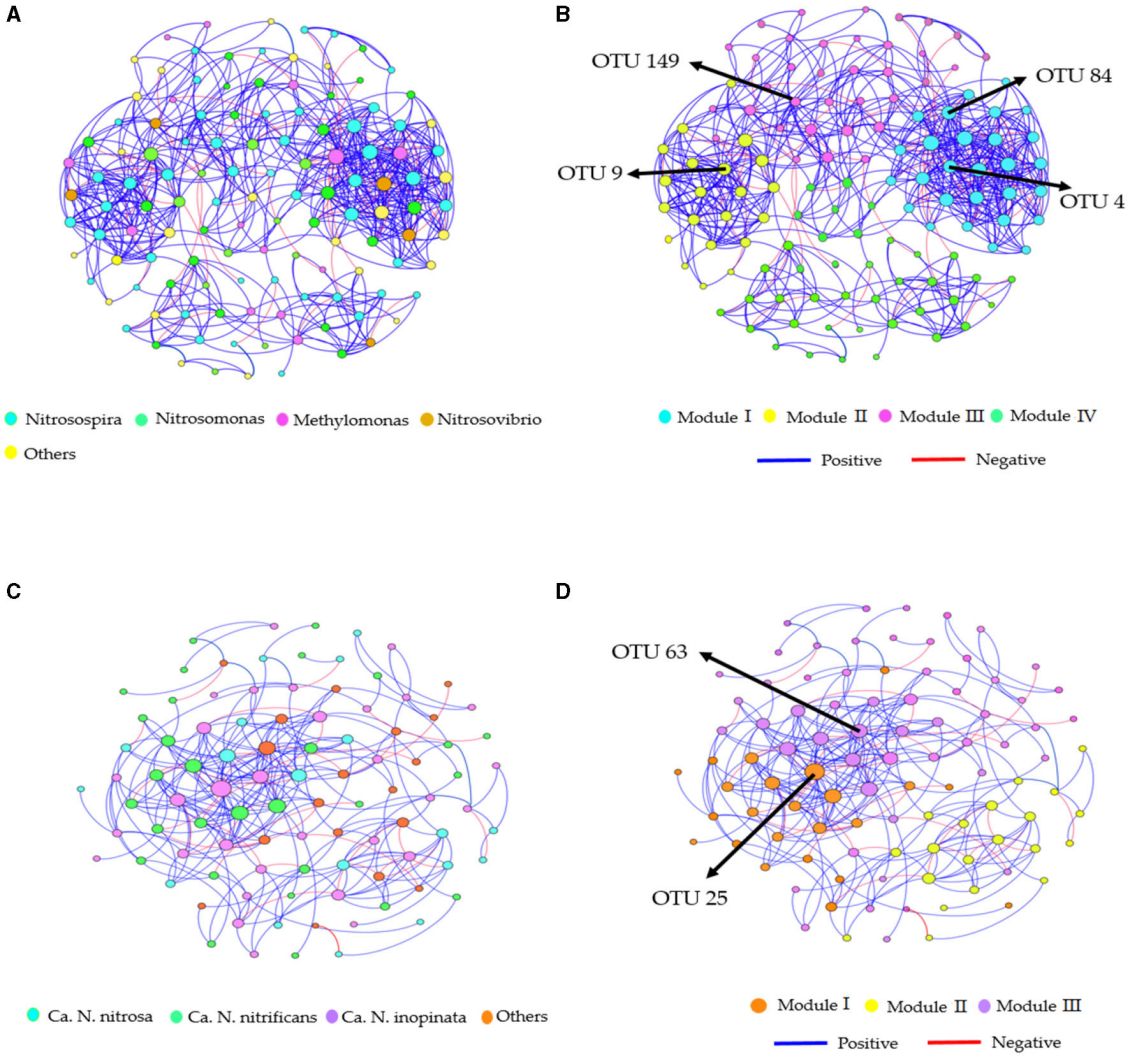
Co-occurrence network analysis of soil nitrification communities at the genus level. **(A, B)** *amoA*–AOB network OTU taxa and modules in the rhizosphere soil; **(C, D)** comammox *Nitrospira* (CAOB) network OTU taxa and modules in the rhizosphere soil. Modules consist of clusters of closely interconnected nodes. The size of the OTU nodes indicates their degrees, and they are colored based on their genus-level classification. Numbers identified in the modules indicate the Keystone taxa. Blue edges represent positive associations, while red edges represent negative associations.

Furthermore, the AOB and CAOB network communities exhibited a superior number of positive associations (411 and 197 edges) than negative associations (186 and 81 edges), respectively (Figures 6A–D). Modules I and III displayed more positive associations (126 and 138 edges) than negative correlations (60 and 67 edges) in the AOB network community (Figures 6A, B). However, the CAOB network community, Modules I and II, expressed a more positive relationship (107 and 51 edges) than a negative relationship (46 and 22 edges) compared to Modules II and IV (Figures 6C, D). Furthermore, key taxonomic groups were identified by assessing the network centrality and closeness centrality across the OTU modules. The AOB community was primarily composed of the genera *Nitrosospira* and *Nitrosomonas*, whereas the CAOB network *community*, genera Ca. *N. inopinata*, Ca. *N. nitrificans* and Ca. *N. nitrosa* (Figures 6B, D).

**Figure 7:**
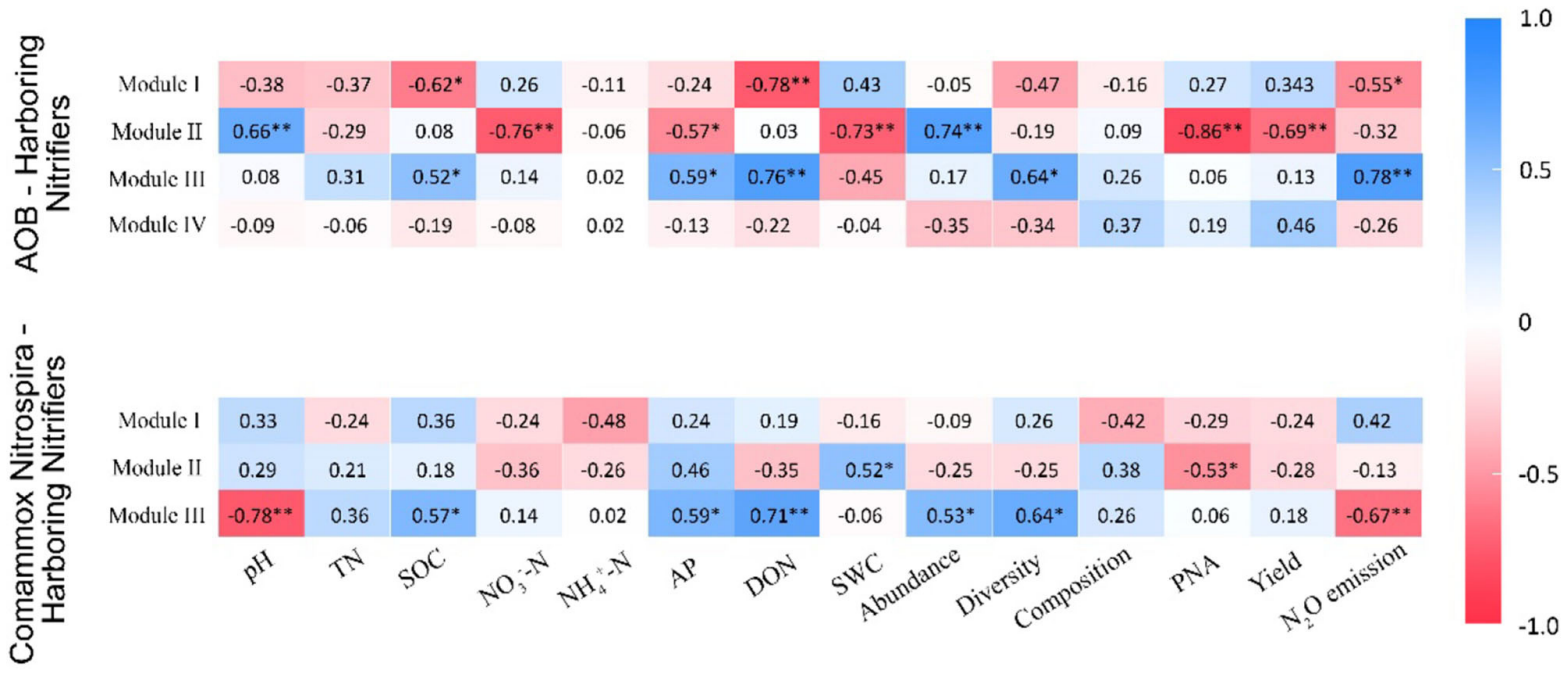
Correlation coefficients between major modules and soil parameters on the *amoA*–AOB and comammox *Nitrospira* (CAOB) nitrifier genes. Red represents positive correlation, and blue represents a negative correlation. The asterisk symbol represents the statistical significance, where *stands for *p* < 0.05 and **for a *p*-value of <0.01. TN, total nitrogen; SWC, soil water content; SOC, soil organic carbon; ${\text{NO}}_{3}^{-}$-N, nitrate nitrogen; ${\text{NH}}_{4}^{+}$-N, ammonia nitrogen; AP, available phosphorus; DON, dissolved organic nitrogen, PNA, potential nitrification activity; yield, sum of grain and biomass and N_2_O emission. The nitrification communities are represented by abundance (the copy numbers of genes), diversity (Chao1 richness), and composition (first principal coordinates, PC1).

In the AOB network, Module I had a negative association with SOC (*r* = −0.62, *p* < 0.05), DON (*r* = −0.78, *p* < 0.01), and N_2_O emissions (*r* = −0.55, *p* < 0.05; Figure 7). Module II was positively correlated with pH (*r* = 0.66, *p* < 0.01) and abundance (*r* = 0.74, *p* < 0.01) but negatively associated with ${\text{NO}}_{3}^{-}$-N (*r* = −0.76, *p* < 0.01), AP (*r* = −0.57, *p* < 0.05), SWC (*r* = −0.73, *p* < 0.01), PNA (*r* = −0.86, *p* < 0.01), and yield (*r* = −0.69, *p* < 0.01; Figure 7). Module III was positively correlated with SOC (*r* = 0.52, *p* < 0.05), AP (*r* = 0.59, *p* < 0.05), DON (*r* = 0.76, *p* < 0.01), diversity (*r* = 0.64, *p* < 0.05), and N_2_O emissions (*r* = 0.75, *p* < 0.01; Figure 7). In the CAOB network, Module II showed a positive correlation to SWC (*r* = 0.52, *p* < 0.05), while it displayed a negative relation with PNA (*r* = −0.53, *p* < 0.05; Figure 7). Module III exhibited a positive association with SOC (*r* = 0.57, *p* < 0.05), AP (*r* = 0.59, *p* < 0.05), DON (*r* = 0.71, *p* < 0.01), abundance (*r* = 0.53, *p* < 0.05), and diversity (*r* = 0.64, *p* < 0.05), but was negatively correlated with pH (*r* = −0.78, *p* < 0.01) and N_2_O emissions (*r* = −0.67, *p* < 0.01; Figure 7).

### Soil properties, AOB, and CAOB communities affected maize productivity, NUE, and N_2_O emission

**Figure 8:**
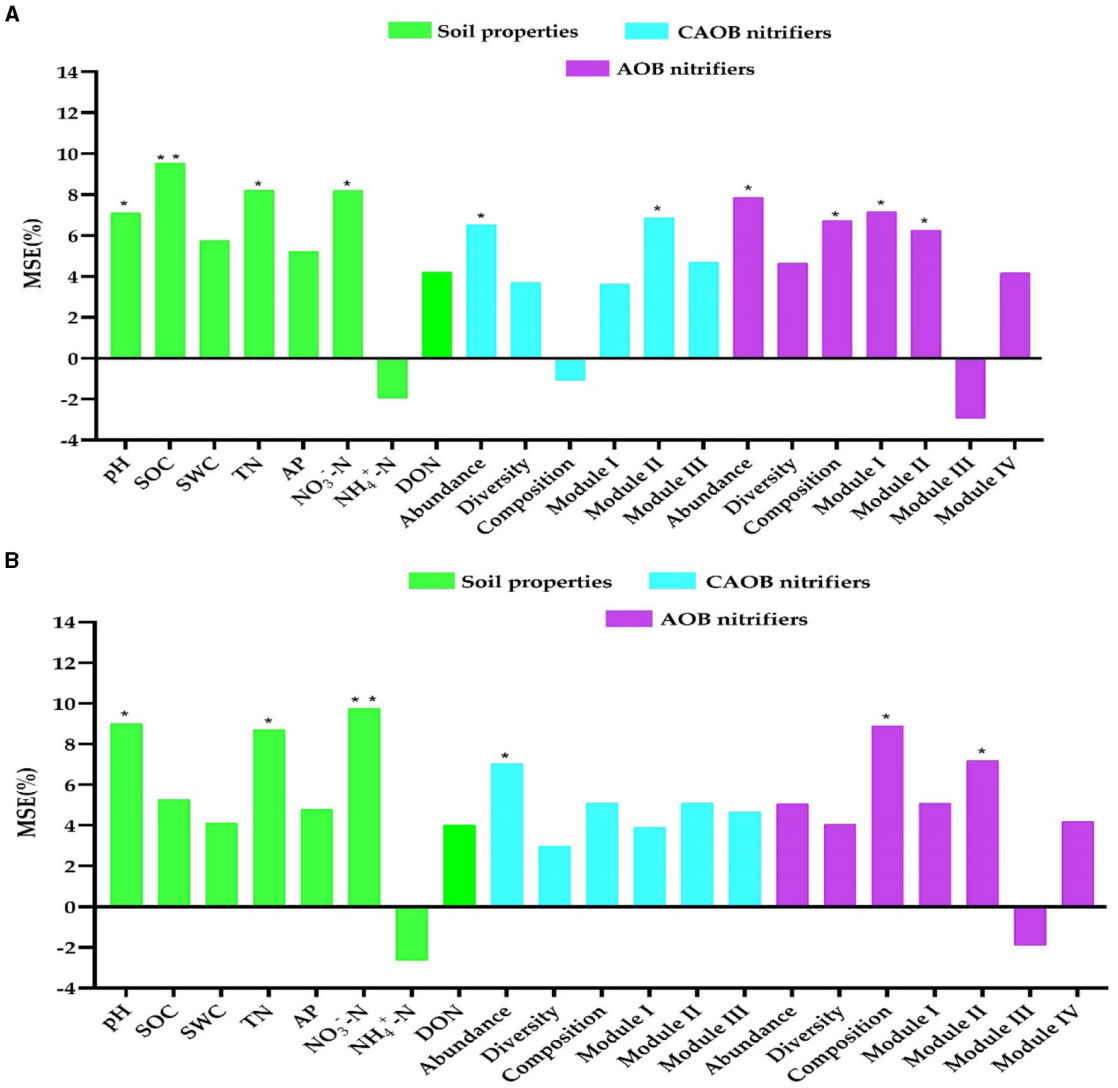
Random forest modeling was performed to evaluate the contributions of soil physiochemical properties and soil nitrification community variables to N_2_O emission and maize productivity. **(A)** N_2_O emission and **(B)** maize productivity in the *amoA*–AOB and comammox *Nitrospira* (CAOB) nitrifiers in the rhizosphere soil. Random forest modeling was performed based on 15 samples (5 treatments × 3 replicates). Soil properties include pH, total nitrogen (TN), soil organic carbon (SOC), available phosphorus (AP), nitrate nitrogen (${\text{NO}}_{3}^{-}$-N), ammonium nitrogen (${\text{NH}}_{4}^{+}$-N), and dissolved organic nitrogen (DON). The soil nitrifying community includes diversity (Shannon index), composition (first principal coordinates, PC1), and three module eigengenes in the trophic co-occurrence network. *p < 0.05; **p < 0.01.

The study utilized random forest modeling to ascertain potential N_2_O emissions, maize productivity, and NUE predictors, including soil properties and the AOB and CAOB populations. Random forest modeling revealed that pH (7.1%, *p* < 0.05 and 9.0%, *p* < 0.05), SOC (9.5%, *p* < 0.01 and 5.3%, *p* > 0.01), TN (8.2%, *p* < 0.05 and 8.7%, *p* < 0.01), and ${\text{NO}}_{3}^{-}$-N (8.1%, *p* < 0.05 and 9.8%, *p* < 0.01) were identified as the crucial indicators of abiotic variables on the N_2_O emission and maize productivity, respectively (Figures 8A, B). Furthermore, the abundance (5.0%−7.8%, *p* < 0.05 and 6.5%−7.0%, *p* < 0.05), composition (6.7%−8.9%, *p* < 0.01 and −1.1%−5.1%, 6.5%, *p* > 0.05), Module I (5.1%−7.1%, *p* < 0.05 and 3.6%−3.9%, *p* < 0.05), and Module II (6.2%−7.2%, *p* < 0.05; and 5.0%−6.9%, *p* < 0.05) were identified as the biotic drivers of the N_2_O emission and maize yield in the AOB and CAOB nitrifiers, respectively (Figures 8A, B).

**Figure 9:**
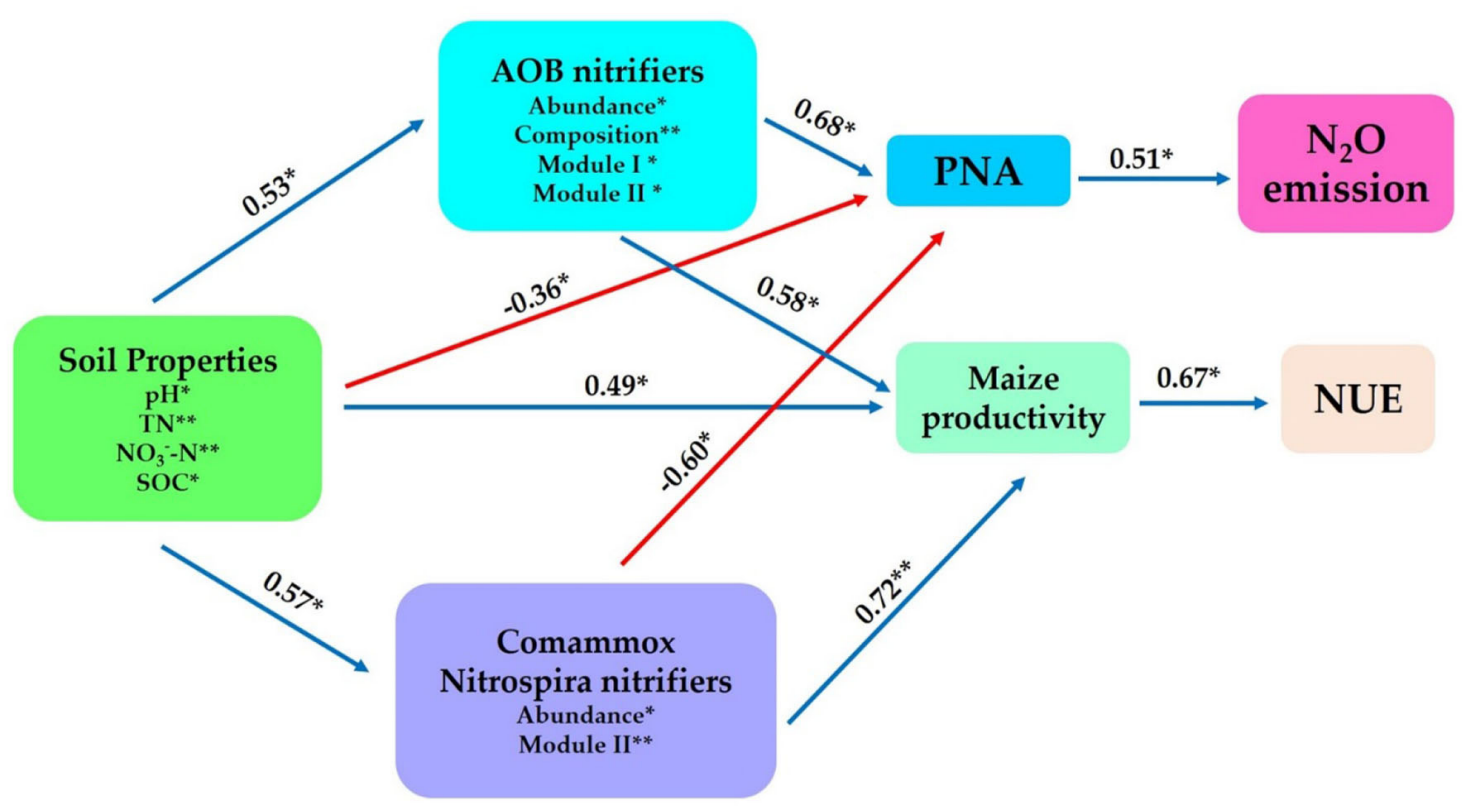
Structural equation modeling was performed to indicate the direct and indirect significant effects of soil physiochemical properties and soil nitrification communities (*amoA*–AOB and comammox *Nitrospira* (CAOB) nitrifiers) on N_2_O emission, maize productivity, and NUE. In rhizosphere soil. Soil properties include pH, total nitrogen (TN), soil organic carbon (SOC), available phosphorus (AP), nitrate nitrogen (${\text{NO}}_{3}^{-}$-N), ammonium nitrogen (${\text{NH}}_{4}^{+}$-N), and dissolved organic nitrogen (DON). The soil nitrifying community includes diversity (Shannon index), composition (first principal coordinates, PC1), and three module eigengenes in the trophic co-occurrence network. *p < 0.05; **p < 0.01.

Structural equation modeling was constructed to further interconnect the prospective predictor's influences of AOB and CAOB nitrifier communities (i.e., composition, abundance, diversity, and network modules) and the abiotic drivers (i.e., soil properties) and their impacts on potential nitrification activity (PNA), the N_2_O emission, maize productivity, and NUE. The physiochemical soil properties (i.e., SOC, pH, TN, and ${\text{NO}}_{3}^{-}$-N) showed significant positive effects on the soil AOB community through abundance, composition, Module I, and Module II (*r* = 0.53, *p* < 0.05), as well as the CAOB community through abundance and Module II (*r* = 0.57, *p* < 0.05) and maize productivity (*r* = 0.49, *p* < 0.05), but expressed a significant negative effect on the PNA (*r* = −0.36, *p* < 0.05; Figure 9).

The AOB community exhibited a significant positive effect on the PNA (*r* = 0.68, *p* < 0.05) and maize productivity (*r* = 0.58, *p* < 0.05) through abundance, composition, Module I, and Module II. On the other hand, the CAOB community, through abundance and Module II, displayed a significant positive effect on maize productivity (*r* = 0.72, *p* < 0.01) but expressed a significantly negative influence with potential nitrification activity (PNA; *r* = −0.60, *p* < 0.05). PNA showed a significant positive association with N_2_O emission (*r* = 0.51, *p* < 0.05). However, maize productivity (*r* = 0.69, *p* < 0.01) exhibited a positive correlation with NUE (*r* = 0.53, *p* < 0.05; Figure 9). The result points to the fact that AOB nitrifiers link positively to PNA activity, which contributes significantly to N_2_O emissions compared to CAOB nitrifier communities. However, soil microbiome diversity and abiotic factors might influence the positive association between maize productivity and NUE.

## Discussion

### Fertilization impacts maize productivity, nitrogen use efficiency, and N_2_O emissions

The application of inorganic fertilizer (CF) and the combined use of inorganic and organic fertilizer (SC) treatments exerted a significant positive influence on maize yield and NUE when compared to alternative treatments. These findings emphasize the pivotal role of appropriate fertilizer management strategies in optimizing agricultural productivity and nutrient utilization (Ouyang et al., 2018; Cardenas et al., 2019). The utilization of inorganic fertilizer (CF) and the combined application of inorganic and organic fertilizer (SC) ensure a continuous supply of nitrogen derived from organic matter. This improved synchronization between the rate of nitrogen uptake by crops and its availability in the soil environment, resulting in enhanced yields and a moderate level of nitrogen use efficiency (Lin et al., 2020; Wang et al., 2020; Govindasamy et al., 2023).

In our study, N_2_O emissions were higher in soils treated with organic fertilizer (MS and SM), with the highest levels observed between June and July. N_2_O emissions are comparatively lower in processed organic fertilizers than in raw organic fertilizers. Additionally, prevailing climatic conditions play a vital role in N_2_O emission rates, as evidenced in the current study. The use of plastic mulch led to an increase in soil moisture and temperature (21–31°C), causing fluctuations in drying and wetting cycles during the same period (June and July). These fluctuations are known to influence N_2_O emissions by altering the populations of nitrifiers (Ouyang et al., 2018; Wang et al., 2020; Chataut et al., 2023). Some studies argue that severe soil water stress conditions can hinder the positive impacts of temperature on nutrient supply to microbes (Prosser and Nicol, 2012; Fowler et al., 2013). The sole application of organic fertilizer treatment significantly increased organic C and improved N_2_O emissions. Existing studies have demonstrated that microorganisms adjust their carbon allocation between cell growth and stress tolerance, impacting their involvement in nutrient cycling (Hink et al., 2018; Lin et al., 2020). The addition of organic fertilizer (SM) and maize straw (MS) creates a conducive environment for nitrifiers due to the high levels of organic carbon and nitrogen compounds. This can enhance soil bacterial functioning and contribute to the production of PNA and nitrous oxide emissions (Shi et al., 2019; Ullah et al., 2020). The CF and SM treatments had a positive effect on the potential nitrification activity (PNA) process relative to the NA treatment. This might be due to the favorable conditions created by inorganic and organic fertilizers for microbial activities in the soil ecosystem (Domeignoz-Horta et al., 2018; Yang et al., 2018; Ullah et al., 2020). The unique niche interactions induced by root exudates promote nitrification processes in alkaline soils within semi-arid regions (Schmidt et al., 2019; Wang et al., 2019). Our research revealed the relationship between higher soil organic carbon (SOC) levels, PNA, and N_2_O emissions when using organic fertilizer. The SC treatment increased soil water-holding capacity, which improved soil aeration (Cui et al., 2016; He et al., 2017). The SC treatment did not significantly affect soil N_2_O emissions relative to solely using inorganic fertilizer (CF). However, the hydrolysis of urea provided highly oxidized nitrate substrates (${\text{NO}}_{3}^{-}$-N) that increased N_2_O emissions (Tao et al., 2017; Ouyang et al., 2018; Fudjoe et al., 2021).

### The AOB community was more active than the CAOB community in response to fertilization

Fluctuations in the abundance and diversity of the soil nitrification community pose a potential threat to the microbiome's function and the long-term sustainability of the soil agroecosystem (Kong et al., 2019; Lin et al., 2020). In our previous study, we found that the AOB, when compared to the abundance and diversity of the AOA, tends not to be sensitive to changes in fertilization regimes (i.e., urea and organic nitrogen fertilizers) and environmental conditions in the semi-arid Loess Plateau. This phenomenon is likely attributed to the mixotrophic lifestyle of AOA (Fudjoe et al., 2021). However, our findings provide compelling evidence that fertilization practices have a substantial impact on the abundance and diversity of the AOB and CAOB communities in the soil. The AOB nitrifiers had a relatively higher influence than CAOB nitrifiers, which can be attributed to their inherent physiological response or a major difference in substrate affinity (Pjevac et al., 2017; Xu et al., 2020).

The AOB were more abundant in the organic (SM) and organic and inorganic (SC) treatments, while the CAOB were higher in the inorganic-treated (CF) soils. Such variations have been linked to the soil's carbon and nitrogen availability (He et al., 2017; Schmidt et al., 2019). Despite the substantial presence of CAOB in all fertilizer treatments, its function and impact on ammonia oxidation were significantly less than those of AOB abundance. This distinction can be attributed to the ecosystem's alkaline soil (Li et al., 2019; Wang et al., 2021). Some studies have traced the reaction to the oligotrophic lifestyle of CAOB, which could make them more sensitive to soils with low levels of nitrogen fertilization (Hu and He, 2017; Kits et al., 2017). Furthermore, these findings validate previous studies indicating that nitrifiers containing AOB are more pivotal in ammonia oxidation and nitrogen cycling within alkaline soils compared to nitrifiers containing CAOB (Hu and He, 2017; Li et al., 2021).

The phylogenetic analysis provides a categorized clustering technique by classifying microbes based on transcription factor lineages and ecological functioning. The *Nitrosospira* cluster was functionally active in the genus AOB community (Hu and He, 2017; Pjevac et al., 2017; Lin et al., 2020). Most of the sequences were associated with *Nitrosospira* cluster 3b, which is affiliated with *N. briensis*, which might be due to the significant nitrogen in alkaline calcareous soils (Wang et al., 2017; Fudjoe et al., 2021). The application of combined inorganic and organic (SC) and sole application of organic (SM and MS) fertilizer relative to no fertilization (NA) might have caused the dominance of *Nitrosospira* Cluster 3b in arable soils due to their nitrification activity (Kong et al., 2019; Wang et al., 2019). Within the genera comammox *Nitrospira*, based on phylogenetic analysis, Clades A.2.1 and A.3 formed the majority of clusters in our analysis, accounting for 89.9% of all sequences relative to *Nitrospira* clade A.1, which accounted for 5.8% of the sequence. In the *Nitrospira* clade A.1, CAOB7 was associated with three key species: Ca. *N. inopinata*, Ca. *N. nitrosa*, and Ca. *N. nitrificans* were significantly higher in the combination of organic and inorganic fertilizer (SC) than the no fertilizer (NA) treatment. The findings of this study are consistent with previous phylogenetic analyses, which have indicated that the majority of comammox *Nitrospira* clade A.1 sequences originate from aquatic or artificial environments, while clades A.2.1 and A.3 are predominantly found in terrestrial ecosystems (Li et al., 2019; Xu et al., 2020). Previous research by Hu and He (2017) reveals that the physiological adaptability and ecological niche differentiation of clades A and B comammox *Nitrospira* can be attributed to the distinct transport proteins and physiological characteristics in each subgroup (He et al., 2017; Pjevac et al., 2017).

### Interactions in the nitrification communities contributed to maize productivity, NUE, and N_2_O emissions

Nitrifying bacteria play a crucial role in regulating nitrogen fixation, N_2_O emissions, and maize productivity in soil. Fertilization practices have a significant impact on the composition and functional diversity of microbial communities involved in decomposition, mineralization, and nitrification processes. Consequently, these modifications in microbial populations influence N_2_O emissions in agricultural fields (Hu and He, 2017; Ouyang et al., 2018). The AOB and CAOB nitrifier communities indicated both positive and significant negative effects on maize productivity, NUE, PNA, and N_2_O emissions, respectively. The current study showed a positive association between AOB and soil property dynamics (soil pH, TN, ${\text{NO}}_{3}^{-}$-N, and SOC), PNA, and N_2_O emission due to potential competitive interactions and functional diversity activities (Lin et al., 2020; Fudjoe et al., 2021; Zheng et al., 2022). On the other hand, the CAOB nitrifier community was negatively correlated with PNA and indirectly with N_2_O emissions but positively associated with maize productivity, NUE, and soil properties (soil pH, TN, ${\text{NO}}_{3}^{-}$-N, and SOC). The positive relationship between the CAOB community and maize NUE might be due to the synergistic effects of nitrogen cycling and nutrient dynamics in the soil ecosystem (Kits et al., 2017; Pjevac et al., 2017). The soil pH, carbon, and nitrogen properties were essential to calcareous soils' microbial cell growth and metabolic activities (Ouyang et al., 2018; Li et al., 2019). The key soil properties are crucial in determining habitat selection and niche differentiation between AOB and CAOB nitrifier communities (Hu and He, 2017; Osburn and Barrett, 2020).

The findings of Berry and Widder (2014) and Mamet et al. (2019) emphasize the significant effect of environmental changes and external factors on the relationships between nitrifying microbes in a community and their ecological functions. The network composition of the AOB and CAOB communities was characterized by more positive correlations between microbes compared to negative correlations (Lin et al., 2020; Fudjoe et al., 2021). The nitrification community's network edges have a high percentage of beneficial interactions compared to adverse connections, which shows that taxonomic competition has increased and enhanced the network structure (Osburn and Barrett, 2020; Zheng et al., 2022). However, the AOB community modules were closely linked to abiotic and biotic variables, unlike the CAOB network populations. The network centrality modules among the different operational taxonomic units (OTUs) demonstrated the importance of interactions and identifying potential keystone taxa (Wang et al., 2020; Li et al., 2021). Keystone taxa are crucial in enhancing competition and maintaining the microbiomes and their functions. Specific keystone taxa and biodiversity adaptability sustained the high diversity of the AOB (*Nitrosospira*) and comammox *Nitrospira* (Ca. *N. inopinata*) communities in response to organic-rich additions (Pjevac et al., 2017; Hink et al., 2018). The soil pH, SOC, ${\text{NO}}_{3}^{-}$-N, and DON sources impacted the keystone taxa of AOB and CAOB nitrifier communities by promoting microbial diversity, community structural integrity, and network stability (Williams et al., 2014; Hu and He, 2017; Lin et al., 2020). Our study further found that the difference in habitat preferences between AOB and CAOB composition was largely due to variations in their cell affinities, mediated keystone taxa performance on nitrogen cycling, and diversity–functioning relationships (Xu et al., 2020; Zheng et al., 2022). Furthermore, discretion is required when concluding on the underlying effect of organic–inorganic fertilization on keystone taxa's considerable impact on nitrification communities (Kong et al., 2019; Li et al., 2019; Schmidt et al., 2019). Additional investigation using stable isotope techniques and empirical data are required to support the current conclusions on the prospective keystone species' contribution to the network system.

## Conclusion

The application of inorganic fertilizer (CF) and the combined use of inorganic and organic fertilizer (SC) resulted in significant improvements in maize productivity and nitrogen use efficiency (NUE) compared to no fertilizer (NA). The SC treatment exhibited lower rates of N_2_O emissions and cumulative N_2_O emissions compared to the CF treatment. This is because SC treatment increased the abundance of comammox *Nitrospira* (CAOB), particularly the clade A.1 lineage group, including Ca. *N. inopinata*, Ca. *N. nitrosa*, and Ca. *N. nitrificans*, compared to the NA treatment. The keystone taxa of the CAOB community showed positive correlations with maize productivity and NUE, likely due to their stimulated functional activities under the SC treatment. Among the AOB community, *Nitrosospira* was the dominant genus at the cluster level; specifically, *N. briensis* species were observed under the CF and SM treatments. The AOB community exhibited a significant positive association with N_2_O emissions and potential nitrification activity (PNA), showing a prominent role in nitrification. Overall, these findings suggest that the SC treatment effectively improves maize yield and nitrogen use efficiency while mitigating N_2_O emissions and promoting a sustainable agroecosystem in the study area.

## Data availability statement

The datasets presented in this study can be found in online repositories. The names of the repository/repositories and accession number(s) can be found at: https://www.ncbi.nlm.nih.gov/genbank/, PRJNA813620 and PRJNA813629.

## Author contributions

SF and LL: conceptualization and methodology. SF: validation, writing—original draft preparation, and visualization. JX and LW: resources. SF: data curation. FY, SA, and LL: writing—review and editing. LL and SS: supervision. JX: project administration. All authors have read and approved the content of the manuscript and agreed to the published version of the manuscript.
