# Supplementary material for: The impact of fertilization on ammonia-oxidizing bacteria and comammox Nitrospira communities and the subsequent effect on N2O emission and maize yield in a semi-arid region
Source: Front Microbiol. 2023 Sep 29;14:1249668. doi: 10.3389/fmicb.2023.1249668 (PMC10570556; doi:10.3389/fmicb.2023.1249668)
Supplement: Supplementary file 1 [file Data_Sheet_1.pdf]

Niche distinction of Ammonia-Oxidizing Bacteria and Comammox-Nitrospira communities in response to fertilizers and the subsequent effect on N_2O emission and Maize yield in a semi-arid region

Setor Kwami Fudjoe[1,2]*, Lingling Li[1,2,]*, Sumera Anwar[3], Shangli Shi[4], Junhong Xie[1,2], Frederick Kwame Yeboah[5], Linlin Wang[1,2]

[1] State Key Laboratory of Aridland Crop Science, Gansu Agricultural University, Lanzhou 730070, China;

[2] College of Agronomy, Gansu Agricultural University, Lanzhou 730070, China;

[3] Department of Botany, Government College Women University Faisalabad, Faisalabad, 38000, Pakistan;

[4] College of Grassland Science, Gansu Agricultural University, Lanzhou 730070, China;

[5] State Key Joint Laboratory of Environment Simulation and Pollution Control, School of Environment, Beijing Normal University, Beijing 100875, China

* **Correspondence:** lill@gsau.edu.cn

Table S.1 Initial physiochemical properties in the soil before experimental setup (2012)

Soil depth (cm)	BD (mg/m3)	pH	TN (g/kg)	AP (g/kg)	SOC (g/kg)
0-5	1.19	8.33	1.05	0.82	9.91
5-10	1.22	8.32	1.05	0.74	8.96
10-30	1.28	8.37	0.94	0.7	8.89

Values are means (n=3). BD= Bulk density; TN=Total Nitrogen; AP= Available Phosphorus; SOC= Soil organic Carbon.

Table S.2 Primer pairs, reaction mixtures, and thermal cycling conditions of qPCR in this study

Target gene	Primer	Primer sequence (5'-3')	Product size (bp)	Amplification condition	Reference
AOB	*amoA*-1F<br>*amoA*-1R	GGGGTTTCTACTGGTGGT<br>CCCCTCKGSAAAGCCTTCTTC	473	94°C, 5 min<br>94°C for 10s, 58°C for 30s, 72°C for 30s, 80°C for 5s, 40 cycles	Fudjoe et al., 2021
CAOB	comaA-244F<br>comaA-659R	GGMATGGTKCCSTGGCA<br>GCCTCGATCAGRTTRTGG	415	94°C, 5 min<br>94°C for 10s, 58°C for 30s, 72°C for 30s, 80°C for 5s, 40 cycles	Li et al., 2021

AOB = Ammonia oxidizing bacteria; *CAOB* = Comammox Nitrospira

PCR Master Mix was purchased from Genepioneer Biotechnologies Co., Nanjing, China.

Table S.3 Effect of fertilization treatments on grain yield, biomass and NUE.

Year	Treatment	Grain yield ($kg\ ha^{-1}$)	Biomass ($kg\ ha^{-1}$)	NUE
2020	NA	4010c	7382c	-
	CF	10551a	15224a	52.8a
	SC	9603a	15195a	48.1b
	SM	5416b	10260b	27.8c
	MS	4201c	8834bc	21.1d
2021	NA	2943c	7551c	-
	CF	8069a	20860a	40.5a
	SC	7575a	19862a	37.9ab
	SM	3499b	13008ab	18.5bc
	MS	3592b	10234b	14.9bc

Values are expressed as mean with different lowercase letters indicating significant differences based on Duncan's HSD test ($p < 0.05$); *, significant at $p < 0.05$; **, significant at $P < 0.01$; ***, significant at $p < 0.001$; NS, not significant at $p < 0.05$. NUE = nitrogen use efficiency. NA, No fertilization; CF, inorganic fertilizer; SC, inorganic plus organic fertilizer; SM, organic fertilizer; MS, maize straw.

TABLE S4 Topological properties of *AOB* and C*AOB*-harboring nitrifier networks in the rhizosphere soil.

Nitrifiers	Module	I	II	III	IV
AOB-harboring nitrifiers	Node	28	21	37	41
	Edge	206	107	186	98
	Average clustering coefficient	0.526	0.428	0.358	0.317
	Average degree	8.609	6.427	8.512	5.136
	Average path length	6.063	7.399	6.124	4.615
	Closeness centrality	0.654	0.482	0.567	0.317
	Network centrality	0.386	0.352	0.416	0.528
	Modularity	0.469	0.327	0.303	0.364
CAOB-harboring nitrifiers	Node	23	20	53	
	Edge	73	52	153	
	Average clustering coefficient	0.205	0.315	0.214	
	Average degree	6.109	5.416	6.879	
	Average path length	5.201	6.486	6.207	
	Closeness centrality	0.163	0.227	0.259	
	Network centrality	0.274	0.258	0.348	
	Modularity	0.369	0.439	0.262	

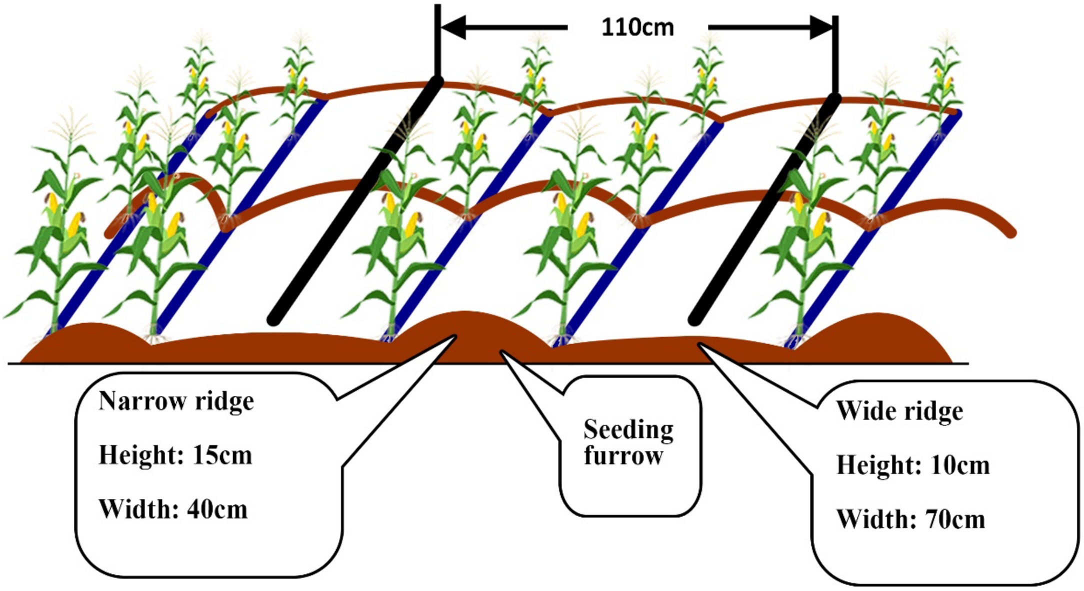

Figure. S.1 An overview of the plot showing ridges

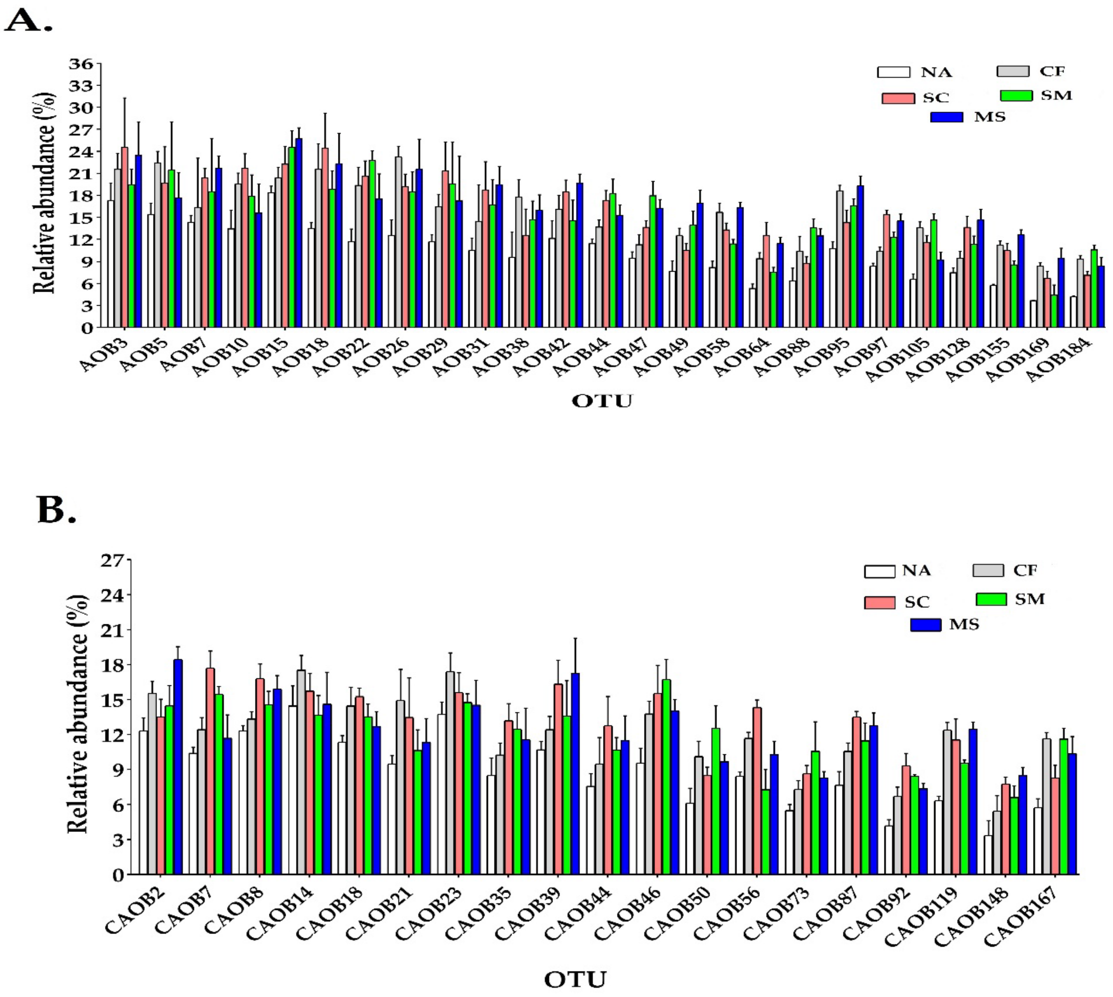

Figure S2 Relative abundances of selected genus cluster of *AOB* (A) *amoA* and *Comammox Nitrospira*-(CAOB) (B) genes in the rhizosphere soil among dominant 200 OTUs, which were significantly changed by N fertilization treatments. Error bars represent standard errors (n = 4). Relative abundance among the N treatments within an OTU (p < 0.05). NA, No fertilization; CF, inorganic fertilizer; SC, inorganic plus organic fertilizer; SM, organic fertilizer; MS, maize straw.

	AOB				Comammox Nitrospira			
	OTU	CHAO	SHANNON	SIMPSON	OTU	CHAO	SHANNON	SIMPSON
pH	**0.54***	-0.38	0.46	0.27	-0.44	**0.61***	-0.32	0.26
TN	0.45	0.27	-0.19	0.32	0.39	0.26	0.29	0.17
SOC	0.29	0.34	**0.52***	0.47	0.42	-0.18	0.27	-0.42
NO_3^--N	0.17	-0.26	0.38	-0.26	-0.28	0.43	0.74*	0.34
NH_4^+-N	-0.29	0.18	-0.32	0.47	0.41	0.29	-0.35	0.42
AP	0.27	0.33	0.41	0.25	0.36	-0.17	-0.48	0.27
DON	-0.15	0.28	**-0.66***	0.22	-0.31	0.19	0.21	-0.38
SWC	-0.34	0.24	0.39	0.27	0.42	-0.32	0.46	**0.52***
PNA	0.21	-0.52*	-0.35	0.19	0.38	0.26	0.41	-0.33
Yield	0.43	0.27	0.31	0.36	-0.27	0.31	-0.39	**-0.57***
N_2O	0.38	0.46	0.15	**0.78***	-0.33	0.46	0.24	-0.43

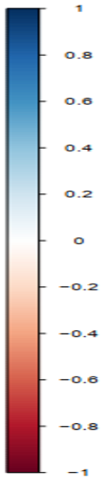

Figure S3 Pearson's correlation between alpha diversity of the bacterial (*AOB*) and *Comammox Nitrospira* (CAOB), soil properties, PNA, N_2O, and yield in the rhizosphere soil. Abbreviations**:** Soil pH; TN = total nitrogen; SOC= soil organic carbon; NO_3^--N= nitrate nitrogen; NH_4^+-N= ammonia nitrogen; AP = available phosphorous; DON = dissolved organic nitrogen; SWC= soil water content; PNA = soil potential nitrification activity; N_2O = cumulative nitrous oxide emission and Yield = sum of grain and biomass. Only significant correlations were indicated; * $p < 0.05$.

REFERENCE

Fudjoe, S.K., Jiang, Y., Li, L., Karikari, B., Xie, J., Wang, L., Anwar, S., Wang, J., 2021. Soil Amendments Alter Ammonia-Oxidizing Archaea and Bacteria Communities in Rain-Fed Maize Field in Semi-Arid Loess Plateau. Land, 10, 10-39.

Li, C., Hu, H. W., Chen, Q. L., Yan, Z. Z., Nguyen, B. A. T., Chen, D., He, J. Z., (2021). Niche specialization of comammox Nitrospira clade A in terrestrial ecosystems. Soil Biology and Biochemistry, 156, 108231. https://doi. org/10.1016/j.soilbio.2021.108231.
